# ChatGPT vs UpToDate: comparative study of usefulness and reliability of Chatbot in common clinical presentations of otorhinolaryngology–head and neck surgery

**DOI:** 10.1007/s00405-023-08423-w

**Published:** 2024-01-13

**Authors:** Ziya Karimov, Irshad Allahverdiyev, Ozlem Yagiz Agayarov, Dogukan Demir, Elvina Almuradova

**Affiliations:** 1 Medicine Program, Ege University Faculty of Medicine, 35100 Izmir, Türkiye; 2 Medicine Program, Istanbul University, Istanbul Faculty of Medicine, Istanbul, Türkiye; 3 Department of Otolaryngology-Head and Neck Surgery, Izmir Tepecik Education and Research Hospital, Health Sciences University, Izmir, Türkiye; 4 Department of Medical Oncology, Ege University Faculty of Medicine, Izmir, Türkiye; 5 Department of Oncology, Medicana International Hospital, Izmir, Türkiye

**Keywords:** Artificial intelligence, Chatbot, ChatGPT, ENT, UpToDate, Otorhinolaryngology and head and neck surgery

## Abstract

**Purpose:**

The usage of Chatbots as a kind of Artificial Intelligence in medicine is getting to increase in recent years. UpToDate® is another well-known search tool established on evidence-based knowledge and is used daily by doctors worldwide. In this study, we aimed to investigate the usefulness and reliability of ChatGPT compared to UpToDate in Otorhinolaryngology and Head and Neck Surgery (ORL–HNS).

**Materials and methods:**

ChatGPT-3.5 and UpToDate were interrogated for the management of 25 common clinical case scenarios (13 males/12 females) recruited from literature considering the daily observation at the Department of Otorhinolaryngology of Ege University Faculty of Medicine. Scientific references for the management were requested for each clinical case. The accuracy of the references in the ChatGPT answers was assessed on a 0–2 scale and the usefulness of the ChatGPT and UpToDate answers was assessed with 1–3 scores by reviewers. UpToDate and ChatGPT 3.5 responses were compared.

**Results:**

ChatGPT did not give references in some questions in contrast to UpToDate. Information on the ChatGPT was limited to 2021. UpToDate supported the paper with subheadings, tables, figures, and algorithms. The mean accuracy score of references in ChatGPT answers was 0.25–weak/unrelated. The median (*Q*1–*Q*3) was 1.00 (1.25–2.00) for ChatGPT and 2.63 (2.75–3.00) for UpToDate, the difference was statistically significant (*p < *0.001). UpToDate was observed more useful and reliable than ChatGPT.

**Conclusions:**

ChatGPT has the potential to support the physicians to find out the information but our results suggest that ChatGPT needs to be improved to increase the usefulness and reliability of medical evidence-based knowledge.

**Supplementary Information:**

The online version contains supplementary material available at 10.1007/s00405-023-08423-w.

## Introduction

The application of Artificial Intelligence (AI) in medicine is getting to increased last decade. Several studies reported the application of AI in clinical grading systems, assessment of cochlear implant function, parathyroid recognition, and prediction of clinical prognosis in otorhinolaryngology–head and neck surgery (ORL–HNS) [1–5]. Ethical concerns such as autonomy, beneficence, nonmaleficence, and justice were emphasized in the paper by Arambula et al. [6].

Chatbots are one of the trending topics of the AI nowadays. ChatGPT (by OpenAI) is one of the most commonly used Chatbots due to the literature. Several studies investigated the application of ChatGPT in medical exams, making a clinical diagnosis, article writing, etc. [4, 7–9].

UpToDate® is a well-known medical knowledge source for physicians that is used in daily clinical practice in worldwide and our hospital [10]. Studies reported its effectiveness on health care quality, decreasing diagnostic error and mortality, association with shorter length of hospital stay, and lower complication rate [11–14]. Another study reported that UpToDate was faster and gave detailed knowledge compared to similar database systems [15].

In this study, we aimed to compare the ChatGPT to UpToDate® for their usefulness and reliability in common clinical presentations of ORL–HNS.

## Materials and methods

### Study design: cross-sectional comparative

#### Study description

ChatGPT version 3.5 [accessed on 27 August 2023 (1–6 cases) and 23 October 2023 (7–25 cases)] and UpToDate® [accessed on 28 August 2023 (1–6 cases) and 23 October 2023 (7–25 cases)] were used for the study. We created 25 case scenarios that are related to the subspecialties of the ORL–HNS. We consider common clinical presentations of the ORL–HNS in the literature while making them [16–23]. These case scenarios include almost equal ratios of the sexes—female/male is 12:13—and different age segments 7 decades of life—of the patients. Clinical presentations are described in Table 1. Then, we asked the ChatGPT “Tell me how would you manage a “number of the age”-year-old male/female patient comes with “... symptoms” that started/for/since day/week/month. Give me references at the end of your response.” and the meantime searched the case on UpToDate.

**Table 1:** Clinical presentations

Case number	Case presentation
1	An 8-year-old male patient comes with a sudden hearing loss that started two days ago
2	A 41-year-old female patient comes with dizziness for a month
3	A 36-year-old male patient comes with recurrent epistaxis
4	A 17-year-old male patient comes with septal deviation and difficulty breathing
5	A 53-year-old female patient comes with snoring during sleep for two months
6	A 26-year-old female patient comes with a painless anterior cervical mass
7	A 22-year-old male patient comes with sneezing, nasal congestion, and, rhinorrhea for 3 days
8	A 33-year-old female patient comes with a runny nose with clear, thin fluid-like water
9	A 14-year-old male patient comes with nasal obstruction, malodorous, and sensation of a foreign body movement within the nose
10	A 55-year-old female patient comes with otalgia for 15 weeks
11	A 66-year-old male patient comes with a painless swelling in the cheek and difficulty in opening the mouth and swallowing
12	A 38-year-old female patient comes with a facial drop in the right that includes the eyelid
13	A 51-year-old male patient comes with ringing in the left ear for one week
14	A 48-year-old female patient comes with a painless, firm, hard thyroid mass for two months
15	A 19-year-old male patient comes with painful swelling in the gingiva since yesterday
16	A 62-year-old female patient comes with nasal obstruction, anosmia, epistaxis, facial pain and swelling, periorbital numbness, and rhinorrhea
17	A 30-year-old male patient is transferred from another rural medical center for consideration of primary hyperparathyroidism as a diagnosis
18	An 18-year-old male patient comes with painless, nonpruritic, bluish, darkly pigmented nodules/plaques on the oral mucosa and face
19	A 69-year-old female patient comes with painless, foul-smelling otorrhea, and conductive hearing loss in the left side
20	A 1-year-old female patient comes with otalgia and fever in the right ear for two days and tender mastoid
21	A 49-year-old male patient comes with dysphonia and difficult breathing for 4 months
22	A 39-year-old female patient comes with anosmia for 6 days
23	A 13-year-old male patient comes with recurrent epistaxis and unilateral nasal obstruction
24	A 2-year-old female patient comes with a fever, trismus, limited cervical neck extension, and dyspnea
25	A 28-year-old male patient comes with preauricular, intermittent, sharp pain, limited jaw motion, and clicking of the temporomandibular joint

We assessed the accuracy of the references in the ChatGPT answers. The scale is: 0—the reference is not available with the described DOI number and source link or is not correct; 1—the reference is available with the described DOI number and source link but not so related to the specific topic; 2—the reference is available with the described DOI number and source link and strongly related to the topic. Then, we calculate the mean score for each answer. In addition, we used the score from 1 to 3 to assess the usefulness of the ChatGPT and UpToDate answers; the scale was reported by Johnson et al. [24]: 1—incomplete answer and not useful; 2—semi-complete answer, somewhat useful but should need some extra knowledge; and 3—complete answer and useful in management.

Afterward, four reviewers assessed each case scenario for ChatGPT answers and related UpToDate papers regarding the search result. Reviewers were blinded to each other’s assessment results.

### Ethical approval

Not applicable to this study because of not include patient data.

### Statistical analysis

The frequencies and percentages were given for categorical variables; and median (IQR: *Q*1–*Q*3) values were given for numerical variables as descriptive statistics. The agreement among the usefulness responses of reviewers for ChatGPT and UpToDate was determined using the coefficients of agreement of “Percent agreement (PA), Fleiss's κ and Gwet AC_1_” [25–27]. All coefficients were presented with 95% confidence intervals (CI). Especially, due to the problems encountered with the Kappa coefficient [28], the Gwet AC_1_ coefficient, which gives more consistent and reliable results, was preferred, but according to the published guide [26], the other two coefficients were also given to present more than one coefficient of agreement. The interpretation of the coefficients was carried out by Gwet's probabilistic method according to the Landis and Koch scale [29]. The McNemar–Bowker test was used to test the symmetry between ChatGPT and UpToDate usefulness responses of each reviewer. In addition, the Wilcoxon rank signed test was used to compare ChatGPT–UpToDate usefulness response means calculated over reviewers.

Statistical significance was assessed at *p < *0.05 and all statistical analyses were performed using R software (R software, version 4.0.5, packages: arsenal-irrcac-ggplot2, R Foundation for Statistical Computing, Vienna, Austria; http://r project.org).

## Results

A comparison of ChatGPT answers to UpToDate search results is described in Appendix 1 in supplementary material.

UpToDate supported its information with references from peer-reviewed journals, conference papers, book chapters, etc. However, ChatGPT did not give references in some questions. The overall mean accuracy score of references in ChatGPT answers was 0.25–weak/unrelated; the mean score of each question was described in Appendix 1 in supplementary material.

**Fig. 1:**
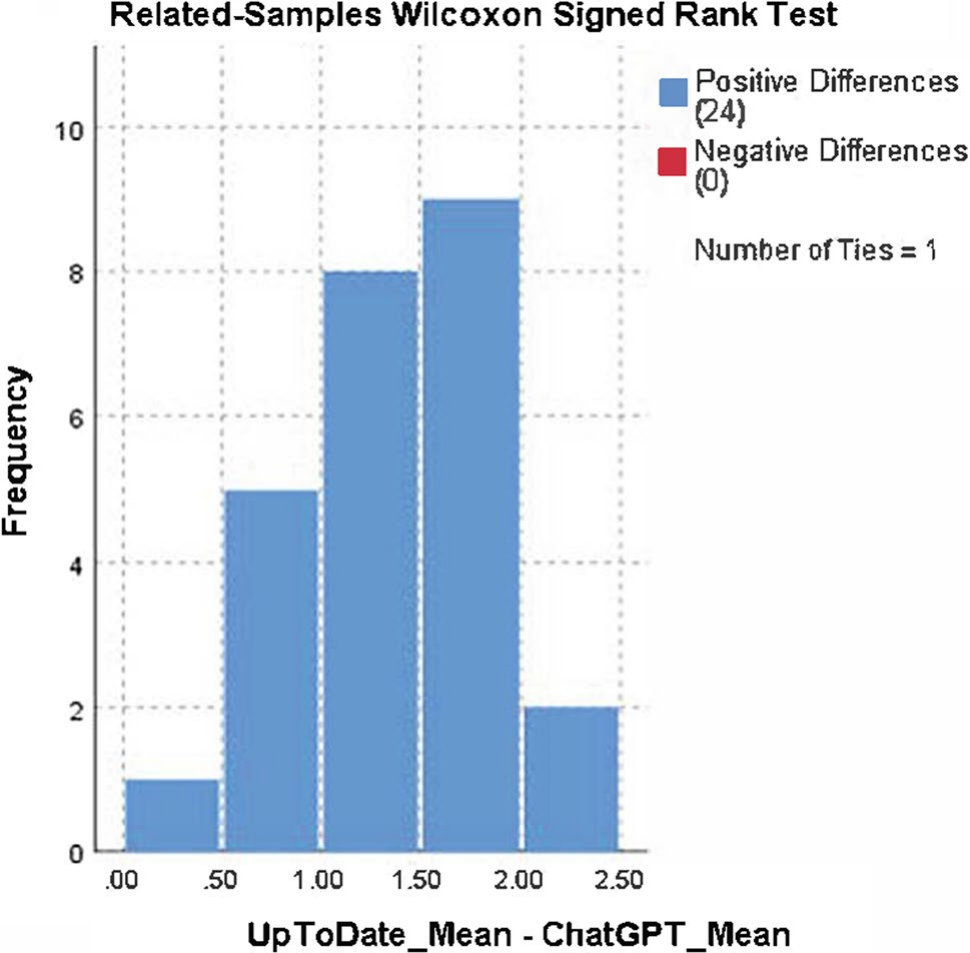
Wilcoxon rank signed test result for comparison of ChatGPT-UpToDate usefulness response means

**Fig. 2:**
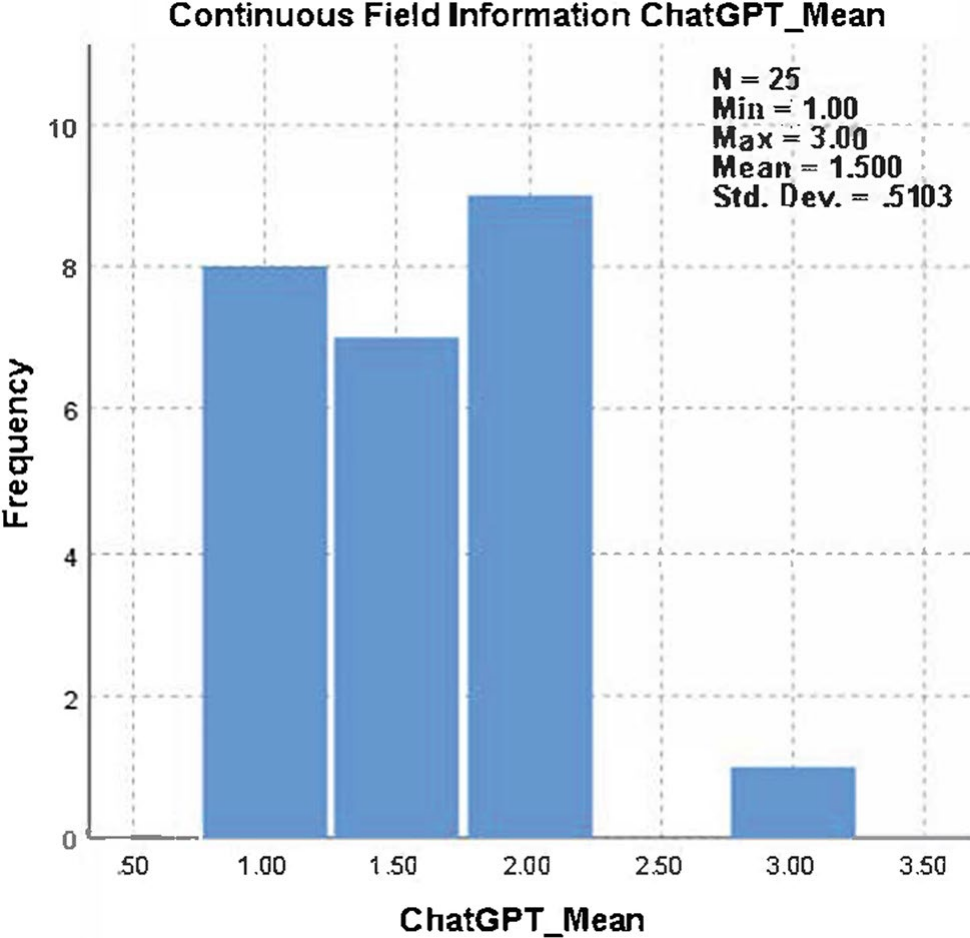
The mean usefulness score distribution for ChatGPT

**Fig. 3:**
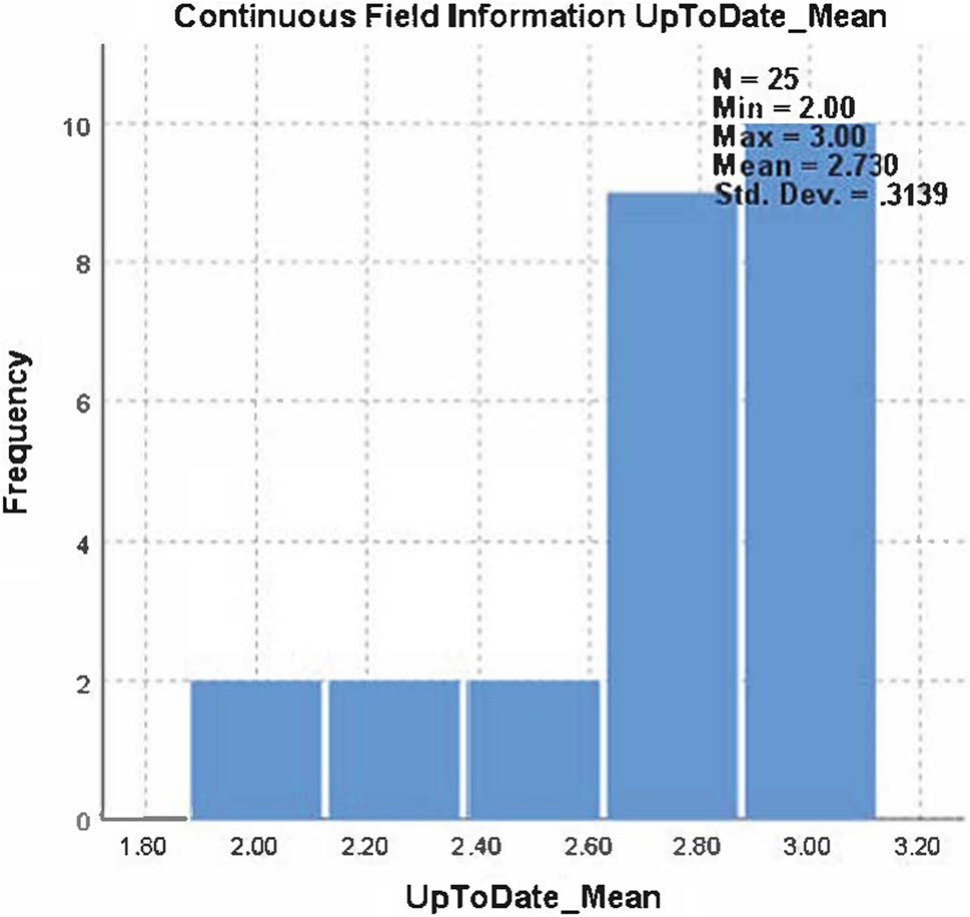
The mean usefulness score distribution for UpToDate

The mean usefulness score was 1.5 ± 0.51 for ChatGPT and 2.73 ± 0.31 for UpToDate. Each reviewer scored the UpToDate responses 2 or 3 points; therefore, UpToDate had a higher overall mean score than ChatGPT. The median (Q1–Q3) was 1.00 (1.25–2.00) for ChatGPT and 2.63 (2.75–3.00) for UpToDate, and the difference was statistically significant (Wilcoxon test, *p < *0.001) (Tables 2, 3 and Fig. 1). When the usefulness scores were compared for two groups for each reviewer, the result was found to be statistically significant (McNmear–Bowker *p* values for each reviewer, *p < *0.001). The mean usefulness score distribution for ChatGPT and UpToDate is also described in Figs. 2 and 3, respectively.

**Table 2:** Distribution of usefulness score in ChatGPT and UpToDate

Usefulness score	ChatGPT	UpToDate	*p* value
	*n* (%)	*n* (%)	
1	54 (54.0%)	0 (0%)	–
2	42 (42.0%)	27 (27.0%)	
3	4 (4.0%)	73 (73.0%)	
Median (*Q*1–*Q*3)	1.00 (1.25–2.00)	2.63 (2.75–3.00)	< 0.001

**Table 3:** Agreement among the usefulness responses of reviewers for ChatGPT and UpToDate

Coefficient	Value	95% CI	Interpretation
ChatGPT			
Gwet's AC_1_	0.86	(0.78–0.93)	Substantial
Percent agreement	0.92	(0.89–0.97)	Almost perfect
Kappa	0.65	(0.41–0.90)	Moderate
UpToDate			
Gwet's AC_1_	0.55	(0.32–0.78)	Fair
Percent agreement	0.73	(0.61–0.84)	Substantial
Kappa	0.30	(0.02–0.60)	Slight

*AC* agreement coefficient

UpToDate supported the topic with algorithms, figures, and tables that are different from ChatGPT. ChatGPT supported many answers by declaring “I am not a doctor” and advising to ask physicians for professional medical advice (highlighted in bold in Appendix 1 in supplementary material). The knowledge by the ChatGPT was extracted from sources with limited to older date, 2021 year (please look at the end of the answer of the first case scenario in Appendix 1 in supplementary material).

## Discussion

The usage of AI in medicine is increasing. Its application to surgical fields has been on trend in recent years. ChatGPT (version 3.5) is a free AI Chatbot and was released by OpenAI at the end of last year. Afterward, it became a trended research topic for doctors and researchers very quickly. Over a thousand article is found in PubMed while searching with the “ChatGPT” keyword right now (accessed on 28 Aug 2023).

There are a limited number of studies evaluating the ChatGPT in ENT&HNS in the literature. Most of them focused the exam-based work. Brennan et al. reported ChatGPT benefit on ear, nose, and throat (ENT) surgical education [30]. Qu et al. evaluated the diagnostic application of ChatGPT and reported the low quality of the Chatbot [4]. Hoch et al. assessed ChatGPT skills in single and multiple choice ENT board questions, and it performed a low correct answer percentage [8]. Other studies evaluated the triage and radiologic diagnosis accuracy of ChatGPT, but the accurate decision ratio was below that of physicians [31, 32]. Ayoub et al. compared the ChatGPT with Google Search and reported the first one had a good result for general medical knowledge but a worse result for medical advice than the second one [33].

UpToDate differs from ChatGPT with a subscription fee—institutional or personal [34]. However, ChatGPT was free access for people when released date, and version 3.5—used in our study—is still free, which makes it useful and reachable for all physicians. However, the upper version requires payment [35]. In addition, ChatGPT can search for more databases/websites and extract knowledge from various sources and languages. UpToDate supports sixteen languages (accessed 28 Aug 2023), but ChatGPT can extract data from more than 25 languages (accessed 28 Aug 2023). The papers’ contents are the same in all languages in UpToDate. However, the answer may change with a wide range of different languages in ChatGPT.

Another nuance is that ChatGPT’s answer depends on the question style and writing format. It requires “well-written” questions to get better answers. We should emphasize that answers to the same question also could result in a wary range depending on the question style. We tried the different versions of the question style and finally unanimously decided on “Tell me how would you manage a “number of the age”-year-old male/female patient comes with “... symptoms” that started/for/since day/week/month. Give me references at the end of your response” format. This nuance is subjective and could be a bias for studies asking open questions to ChatGPT like our study. When we decided to question format, we considered the details of the answers, and in addition, asked for references to improve sources. Because, when we asked ChatGPT a question without the phrase “Give me references at the end of your response”, it did not give any references. Therefore, if a physician wants to get a reference to find out more information related to the topic, he/she should write an extra sentence while asking the question. This decreases the usefulness and reliability of the ChatGPT. Supporting the knowledge with references from peer-reviewed journals, conference papers, and book chapters increases the reliability and makes the knowledge transparent in UpToDate. Besides, promoting the topic with algorithms, figures, and tables makes UpToDate more systematic and beneficial.

UpToDate’s search tool finds the related paper from its database regarding the search keywords. However, ChatGPT searches for many websites and databases. Papers in UpToDate included main subheadings that ease the physician's work to find the wanted information quickly within the paper. In addition, ChatGPT gave a subheading while asking about the management of patients, however, this heading contains non-specific sentences. Therefore, it looks like a useful feature of UpToDate. On the contrary, ChatGPT replies to the questions quickly differ from UpToDate and decrease the time to reach out for knowledge. It is one of the strong features of ChatGPT. UpToDate requires finding related papers and headings/subheadings within the papers manually and takes time.

ChatGPT’s information base is limited to 2021 due to its training; therefore, it is a weak feature of Chatbot regarding further and most updated knowledge [33]. In addition, we observed the same result while looking at the references of Chatbot’s answers. ChatGPT emphasized that in some answers reference parts its last knowledge was updated in September 2021. Informing the users on this issue is a good point regarding ethics. On the other hand, medical knowledge in UpToDate is reviewed and updated by doctors, well-experienced specialists, and academicians continuously.

Interestingly, ChatGPT cited and recommended the UpToDate while answering our questions in the 7th and 25th cases.

It was observed that ChatGPT give medical recommendation in contrast to basic medical knowledge in the reported studies ^33^. This is an important concern for the safety of patients. In our study, we did not observe it. In addition, UpToDate gives medical recommendations, but these are evidence-based and supported by studies. In our study, most of the references in ChatGPT answers were unrelated to the question and some of them were inaccessible/unavailable. ChatGPT supported many answers by declaring “I am not a doctor” and advises referral to physicians for professional medical advice. This is a good point for ethical issues related to the AI. In addition, repeating sentences in the same answer in ChatGPT may be wordy while reading.

ChatGPT’s answers may vary on different computers, in different locations, and at different times. The questions in our paper were answered differently according to this issue. We used the same computer device for asking the question to ChatGPT.

Twenty-five clinical case scenarios were investigated in the study which is a limited number. ChatGPT summarized the result itself, but we searched and selected the appropriate monograph in the UpToDate. Hence, it is a subjective factor of the authors’ selection. Because there are several monographs for the same search result in the UpToDate. In this study, UpToDate had more usefulness scores and reliability than ChatGPT with statistical significance.

## Conclusion

In this study, we aimed to investigate the usefulness and reliability of ChatGPT in comparison with UpToDate in common clinical presentations of otorhinolaryngology–head and neck surgery. In this stage, UpToDate looks more useful and reliable than ChatGPT. Developers need to improve the ChatGPT with evidence-based search and analysis skills and update its database.

### Supplementary Information

Below is the link to the electronic supplementary material.

Supplementary file1 (DOCX 68 KB)

## Data Availability

Not applicable.
