# Supplementary material for: ChatGPT vs UpToDate: comparative study of usefulness and reliability of Chatbot in common clinical presentations of otorhinolaryngology–head and neck surgery
Source: Eur Arch Otorhinolaryngol. 2024 Jan 13;281(4):2145–51. doi: 10.1007/s00405-023-08423-w (PMC10942922; doi:10.1007/s00405-023-08423-w)
Supplement: Supplementary file 1 — Supplementary file1 (DOCX 68 KB) [file 405_2023_8423_MOESM1_ESM.docx]

**Appendix 1:** Comparison of ChatGPT answers to papers on the related subject in UpToDate.

| *ChatGPT:* | *UpToDate:* |
| --- | --- |
| *1. Case* | |
| Question: Tell me how would you manage an 8-year-old male patient who comes with a sudden hearing loss that started two days ago. Give me references at the end of your response.  **[** Answer: **I am not a doctor**, but I can offer some general information on how an 8-year-old male patient presenting with sudden hearing loss might be managed. However, **it's important to consult a medical professional** for accurate advice tailored to the specific situation. Sudden hearing loss in children can have various causes, including infections, trauma, and other underlying conditions. Here's a possible approach to managing this case:  1. Medical History and Physical Examination: Begin by taking a detailed medical history, including any recent illnesses, exposure to loud noises, or head trauma. Conduct a thorough physical examination, paying attention to the ears, nose, and throat, to identify any signs of infection or injury.  2. Audiometric Testing: Perform audiometric testing, such as pure-tone audiometry and tympanometry, to assess the extent and type of hearing loss. This will help determine whether the hearing loss is conductive, sensorineural, or mixed.  3. Otoscopic Examination: Examine the child's ear canals and eardrums using an otoscope to check for any signs of inflammation, infection, or blockages that might be causing the hearing loss.  4. Laboratory Tests: Depending on the findings from the history and physical examination, additional laboratory tests might be necessary. These could include blood tests to check for signs of infection or inflammation.  5. Imaging Studies: In some cases, imaging studies like MRI or CT scans might be required to investigate any potential structural abnormalities in the ear or the auditory pathways.  6. Treatment: The treatment plan will depend on the underlying cause of the sudden hearing loss. If an infection is identified, appropriate antibiotics or antiviral medications might be prescribed. For sensorineural hearing loss, management strategies could involve hearing aids or cochlear implants, if appropriate. Steroid therapy might be considered in cases of sudden sensorineural hearing loss of unknown origin.  7. Follow-Up: Schedule regular follow-up appointments to monitor the child's progress and adjust the treatment plan if needed.  8. Referral: Depending on the findings and progress, the child might need to be referred to an audiologist, otolaryngologist (ear, nose, and throat specialist), or other relevant specialists.  9. Educating the Family: Provide the child's family with information about the diagnosis, treatment plan, and potential outcomes. Educate them about preventive measures, if applicable (e.g., protecting the ears from loud noises).  10. Emotional Support: Sudden hearing loss can be distressing for both the child and the family. Offer emotional support and resources to help them cope with the situation.  Remember that this is a general guideline and not a substitute for professional medical advice. Always consult a qualified healthcare provider for accurate diagnosis and treatment.  References:  **Please note that I don't have direct access to external references. The information provided is based on general medical knowledge up to September 2021.** For up-to-date and accurate information, consult medical literature and resources such as:  1. American Academy of Pediatrics. (2016). Guidelines for Hearing Screening After the Newborn Period to Kindergarten Age: Clinical Practice Guideline—Executive Summary. Pediatrics, 138(2), e20162161. doi:10.1542/peds.2016-2161  2. American Speech-Language-Hearing Association. (2021). Childhood Hearing Loss: Identification and Intervention. Retrieved from https://www.asha.org/public/hearing/Childhood-Hearing-Loss/  3. Shargorodsky, J., Curhan, S. G., & Curhan, G. C. (2010). A Prospective Study of Vitamin Intake and the Risk of Hearing Loss in Men. The American Journal of Clinical Nutrition, 92(2), 416–421. doi:10.3945/ajcn.2010.29674  Remember to consult with a healthcare professional for personalized advice and management. **]**  **The mean accuracy score of references: 0** | Smith, Richard JH, and Adrian Gooi. “Hearing loss in children: screening and evaluation.”  Text link: <https://www.uptodate.com/contents/hearing-loss-in-children-screening-and-evaluation?search=sudden%20hearing%20loss%20in%20children&source=search_result&selectedTitle=1~150&usage_type=default&display_rank=1>  Topic 6297 Version 32.0 |
| *2. Case* | |
| Question: Tell me how would you manage a 41-year-old female patient who comes with dizziness for a month. Give me references at the end of your response.  **[**Answer: **I'm not a doctor**, but I can offer you a general outline of how a healthcare professional might approach the case of a 41-year-old female patient presenting with dizziness for a month. It's important to note that individual cases can vary, and a **qualified medical professional would be best suited to provide accurate advice** based on a thorough evaluation of the patient. Here's a potential approach:  1. Medical History and Initial Assessment:  Start by taking a detailed medical history, including the onset, duration, and characteristics of the dizziness, any associated symptoms (such as nausea, vomiting, hearing loss, or palpitations), any recent illnesses, medications, or changes in lifestyle. Perform a physical examination to check for signs of any neurological or cardiovascular abnormalities.  2. Review of Medications and Medical Conditions:  Review the patient's current medications and medical conditions to identify any potential drug interactions or underlying conditions that could contribute to dizziness.  3. Differential Diagnosis:  Dizziness can stem from various causes, including inner ear disorders (like benign paroxysmal positional vertigo), vestibular migraines, Meniere's disease, vestibular neuritis, orthostatic hypotension, cardiovascular issues, anxiety or panic disorders, and more. The medical history and physical examination findings will guide the clinician toward a focused differential diagnosis.  4. Additional Testing:  Depending on the initial assessment, further tests may be warranted. These could include:  - Audiometry and vestibular testing to assess inner ear function.  - Blood tests to evaluate electrolyte imbalances, anemia, or other metabolic causes.  - Electrocardiogram (ECG) to rule out cardiac arrhythmias.  - Imaging studies (e.g., MRI or CT scan) to assess the brain and inner ear structures.  5. Referral to Specialists:  If the cause remains unclear or if the dizziness is related to a specific condition, referral to specialists such as an otolaryngologist, neurologist, or cardiologist may be necessary for further evaluation and management.  6. Treatment Plan:  Treatment will depend on the underlying cause identified through assessment. This could involve:  - Canalith repositioning maneuvers for benign paroxysmal positional vertigo.  - Medications for migraines, if vestibular migraines are suspected.  - Lifestyle modifications for Meniere's disease.  - Physical therapy or vestibular rehabilitation for certain vestibular disorders.  - Medications or interventions to manage cardiac or anxiety-related causes.  7. Follow-up:  Schedule follow-up appointments to monitor the patient's progress and adjust the treatment plan as needed. It's important to provide the patient with clear instructions and education on managing their symptoms.  Remember, **this is a general outline and not a substitute for professional medical advice.** **Always consult a qualified healthcare provider** for accurate diagnosis and treatment recommendations.  References:  - Strupp, M., Brandt, T. (2009). Diagnosis and treatment of vertigo and dizziness. Deutsches Ärzteblatt International, 106(29), 487-495. [DOI:10.3238/arztebl.2009.0487](https://www.aerzteblatt.de/int/archive/article?id=63597)  - Bhattacharyya, N. (2018). The prevalence of benign paroxysmal positional vertigo and associated dizziness in children. International Journal of Pediatric Otorhinolaryngology, 104, 125-128. [DOI:10.1016/j.ijporl.2017.12.025](https://www.sciencedirect.com/science/article/pii/S0165587617311195)  - Baloh, R. W. (2019). Vestibular neuritis. New England Journal of Medicine, 381(22), 2156-2164. [DOI:10.1056/NEJMcp1810660](<https://www.nejm.org/doi/full/10.1056/NEJMcp1810660>) **]**  **The mean accuracy score of references: 0** | Branch, W. T., and J. J. Barton. “Approach to the patient with dizziness.”  Text link: <https://www.uptodate.com/contents/approach-to-the-patient-with-dizziness?search=dizziness&source=search_result&selectedTitle=1~150&usage_type=default&display_rank=1>  Topic 5099 Version 15.0 |
| *3. Case* | |
| Question**:** Tell me how would you manage a 36-year-old male who comes with recurrent epistaxis. Give me references at the end of your response.  **[** Answer: I can provide you with a general outline of how to manage a 36-year-old male who presents with recurrent epistaxis (nosebleeds). However, **please note that I'm not a substitute for professional medical advice, and it's important to consult a qualified healthcare provider for personalized guidance.**  Initial Assessment and History:  1. History Taking: Begin by obtaining a detailed medical history, including any previous episodes of epistaxis, their frequency, duration, severity, and any precipitating factors. Inquire about the patient's medical conditions, medications, allergies, and family history of bleeding disorders.  2. Physical Examination: Conduct a thorough physical examination, paying particular attention to the nasal cavity, septum, and other signs of bleeding disorders. Check blood pressure and assess for signs of anemia.  Diagnostic Workup:  1. Laboratory Tests: Perform relevant laboratory tests to assess coagulation factors, including a complete blood count (CBC), prothrombin time (PT), activated partial thromboplastin time (aPTT), and platelet count. If there's a suspicion of an underlying bleeding disorder, additional tests such as von Willebrand factor assay and clotting factor assays may be considered.  2. Imaging: If indicated, consider imaging studies like nasal endoscopy or CT angiography to identify any anatomical abnormalities or vascular malformations contributing to the recurrent epistaxis.  Management:  1. Immediate Measures:  - Have the patient sit up and lean forward slightly to prevent blood from flowing down the throat.  - Apply direct pressure by pinching the soft part of the nose against the septum for about 10-15 minutes.  - If bleeding persists, use a nasal decongestant spray (e.g., oxymetazoline) to constrict blood vessels and facilitate clotting.  2. Topical Treatments:  - Recommend the use of saline nasal sprays to keep the nasal passages moist and prevent dryness that may contribute to bleeding.  - Advise against inserting objects into the nose, as this can worsen bleeding and cause trauma.  3. Addressing Underlying Causes:  - If the patient has an underlying medical condition contributing to the epistaxis (e.g., hypertension, coagulation disorder), initiate appropriate treatment and management.  - For patients with frequent epistaxis due to nasal dryness, advise using humidifiers, avoiding irritants, and using saline-based moisturizers.  4. Surgical Intervention:  - If structural abnormalities or vascular malformations are identified, the patient may require surgical intervention, such as cauterization, packing, or more advanced procedures.  5. Referral to Specialists:  - Consider referring the patient to an otolaryngologist (ENT specialist) or a hematologist if the recurrent epistaxis is severe, persistent, or indicative of an underlying bleeding disorder.  Patient Education:  - Educate the patient about proper nasal care, including avoiding excessive nose picking, using humidifiers, and using saline sprays.  - Discuss warning signs that require immediate medical attention, such as prolonged bleeding, difficulty breathing, or signs of anemia.  References:  (Please note that I cannot provide direct links to specific references, but you can search for these titles in medical databases or journals.)  1. Ishman SL, Schwab RJ. Epistaxis. In: Kountakis SE, Senior BA, Draf W, et al., editors. The Frontal Sinus. Springer; 2005. p. 139-147.  2. Blumenfeld L, Clinch T. Management of epistaxis. Am Fam Physician. 2005;71(2):305-311.  3. Kucik CJ, Clenney T. Management of epistaxis. Am Fam Physician. 2005;71(2):305-311.  4. Guldfred LA, Lyhne D, Reibel J, et al. Topical hemostatic treatment of epistaxis in hereditary hemorrhagic telangiectasia: a randomized, placebo-controlled, double-blind trial. Laryngoscope. 2014;124(4):973-978.  5. Wormald R, Athanasiadis T, Rees G, et al. The role of endoscopic sinus surgery in the management of adult chronic maxillary sinusitis. Am J Rhinol. 2005;19(4):358-365.  **Remember, it's important to consult with a medical professional** for appropriate assessment and management based on the individual patient's condition. **]**  **The mean accuracy score of references: 0.4** | Alter, Harrison, et al. “Approach to the adult with epistaxis.”  Text link:  <https://www.uptodate.com/contents/approach-to-the-adult-with-epistaxis?search=adult%20with%20epistaxis&source=search_result&selectedTitle=1~150&usage_type=default&display_rank=1>  Topic 265 Version 38.0 |
| *4. Case* | |
| Question: Tell me how would you manage a 17-year-old male patient who comes with septal deviation and difficulty breathing. Give me references at the end of your response.  **[** Answer: **I'm not a doctor**, but I can offer some general information on how a 17-year-old male patient with septal deviation and difficulty breathing might be managed. **It's important to consult a qualified medical professional** for accurate advice tailored to the specific situation. That being said, here's a general outline of potential management steps:  1. Medical History and Physical Examination: The first step would be to gather a detailed medical history, including any prior respiratory issues, allergies, or surgeries. A thorough physical examination, including nasal endoscopy, would help assess the extent of septal deviation and any associated problems.  2. Imaging Studies: Imaging studies such as a CT scan or MRI might be recommended to provide a clear visualization of the nasal structures and the severity of septal deviation.  3. Nonsurgical Management: Depending on the severity of the deviation and associated symptoms, conservative measures like nasal saline irrigation, humidification, and allergy management might be recommended to alleviate breathing difficulties temporarily.  4. Surgical Intervention: If the deviation is causing significant breathing problems, surgical intervention may be considered. Septoplasty is a common surgical procedure aimed at straightening the nasal septum to improve airflow. This procedure can be performed under local or general anesthesia.  5. Postoperative Care: After surgery, postoperative care instructions are crucial for a successful recovery. This may involve nasal packing, pain management, and proper wound care.  6. Follow-up: Regular follow-up appointments will help monitor the patient's healing progress, address any concerns, and ensure that the desired outcomes are achieved.  7. Patient Education: Educating the patient about the condition, treatment options, and postoperative care is essential for their cooperation and understanding throughout the process.  Remember, this is a general guideline and the actual management plan will depend on the individual patient's medical history, the severity of the septal deviation, and the recommendations of a qualified medical professional.  As for references, since I can't provide direct links, here are some potential sources you could consult for more information:  1. American Academy of Otolaryngology - Head and Neck Surgery. (2021). Septoplasty. Retrieved from https://www.entnet.org/content/septoplasty  2. Bhandarkar, N. D., Smith, T. L., Handley, J. M., & Reardon, E. (2010). Septoplasty and turbinate surgery. Otolaryngologic Clinics of North America, 43(4), 897-908. doi:10.1016/j.otc.2010.04.010  3. Svider, P. F., Pashkova, A. A., & Folbe, A. J. (2013). Nasal septal surgery: Indications and techniques. The Ochsner Journal, 13(3), 418-424. PMID: 24052759  **Please consult a healthcare professional** or a medical guideline for the most up-to-date and accurate information regarding the management of septal deviation in a 17-year-old patient. **]**  **The mean accuracy score of references: 0** | “Mendez, Donna Reyes, Annie Lapointe, and Glenn C. Isaacson. “Nasal trauma and fractures in children and adolescents.”  Text link:  <https://www.uptodate.com/contents/nasal-trauma-and-fractures-in-children-and-adolescents?search=septal%20deviation%20and%20difficulty%20breathing%20in%20adolescent&source=search_result&selectedTitle=1~150&usage_type=default&display_rank=1#H10> Topic 6558 Version 26.0 |
| *5. Case* | |
| Question: Tell me how would you manage a 53-year-old female patient who comes with snoring during sleep for two months. Give me references at the end of your response.  **[** Answer: Managing a 53-year-old female patient who presents with snoring during sleep for two months requires a comprehensive approach to diagnose the underlying cause and develop an appropriate treatment plan. Here's a step-by-step outline of how you might manage this patient:  1. History and Physical Examination: Start by taking a detailed medical history, including any relevant medical conditions, medications, allergies, and lifestyle factors. Inquire about the patient's sleep habits, duration of snoring, any witnessed breathing pauses during sleep, daytime sleepiness, and the impact on the patient's quality of life. Perform a thorough physical examination, focusing on the head and neck region, including the nasal passages, oral cavity, tonsils, and uvula.  2. Sleep Diary and Questionnaires: Ask the patient to maintain a sleep diary for at least a week, recording information about bedtime, wake time, number of awakenings, snoring intensity, and daytime symptoms. Additionally, have the patient complete validated sleep questionnaires such as the Epworth Sleepiness Scale (ESS) to assess daytime sleepiness and the Berlin Questionnaire to screen for obstructive sleep apnea (OSA) risk.  3. Sleep Study (Polysomnography): If OSA is suspected, consider ordering a full overnight polysomnography (PSG) study, which can be conducted at a sleep clinic or using portable home sleep testing devices. PSG will help diagnose the severity of sleep apnea, assess the patient's overall sleep architecture, and identify any other sleep disorders.  4. Referral to Specialists: Depending on the findings, you might consider referring the patient to specialists such as an otolaryngologist (ENT) for evaluation of upper airway obstruction, a pulmonologist for respiratory issues, or a sleep medicine specialist for further evaluation and management.  5. Lifestyle Modifications: Encourage the patient to adopt healthy lifestyle changes that can alleviate snoring and improve sleep quality. These may include weight loss (if applicable), avoiding alcohol and sedatives close to bedtime, maintaining a regular sleep schedule, and sleeping on their side instead of their back.  6. Continuous Positive Airway Pressure (CPAP): If OSA is diagnosed, moderate to severe cases may benefit from CPAP therapy. This involves using a machine that delivers a constant stream of air through a mask, keeping the airway open during sleep. Proper fitting of the mask and patient education are essential for compliance.  7. Oral Appliances: For patients with mild to moderate OSA or snoring, oral appliances (mandibular advancement devices) can be considered. These devices reposition the lower jaw and tongue to keep the airway open during sleep.  8. Surgical Options: If anatomical abnormalities such as enlarged tonsils or a deviated septum are contributing to the snoring, surgical interventions like tonsillectomy or septoplasty might be recommended. Surgical options are typically considered after a thorough evaluation by an ENT specialist.  9. Follow-up and Monitoring: Regular follow-up appointments are crucial to assess the patient's response to treatment, address any concerns, and make necessary adjustments to therapy.  10. Patient Education: Provide detailed education about the diagnosed sleep disorder, the chosen treatment approach, and the importance of compliance. Emphasize the potential impact of untreated sleep disorders on overall health.  Remember that the management plan should be tailored to the patient's individual needs, preferences, and medical condition. **Collaborate closely with specialists** to ensure a comprehensive and effective approach to managing the patient's snoring and related sleep issues.  References:  •American Academy of Sleep Medicine. (2014). International Classification of Sleep Disorders (3rd ed.).  •American Academy of Sleep Medicine. (2017). Clinical Practice Guideline for Diagnostic Testing for Adult Obstructive Sleep Apnea: An American Academy of Sleep Medicine Clinical Practice Guideline.  •Kushida, C. A., et al. (2017). Clinical Guidelines for the Manual Titration of Positive Airway Pressure in Patients with Obstructive Sleep Apnea.  •Aurora, R. N., et al. (2010). The Treatment of Central Sleep Apnea Syndromes in Adults: Practice Parameters with an Evidence-Based Literature Review and Meta-Analyses.  •Epstein, L. J., et al. (2009). Clinical Guideline for the Evaluation, Management and Long-term Care of Obstructive Sleep Apnea in Adults.  •American Academy of Otolaryngology–Head and Neck Surgery. (2017). Clinical Practice Guideline: Polysomnography for Sleep-Disordered Breathing Prior to Tonsillectomy in Children.  •Ngiam, J., & Balasubramaniam, R. (2020). Surgical and Medical Treatment Options for Snoring and Obstructive Sleep Apnea.  Please note that these references are based on guidelines available up to September 2021, and there might have been updates or changes since then. Always refer to the most current medical literature and guidelines for the latest recommendations. **]**  **The mean accuracy score of references: 1** | Rowley, James A. “Snoring in adults.”  Text link: <https://www.uptodate.com/contents/snoring-in-adults?search=Snoring%20in%20adults&source=search_result&selectedTitle=1~150&usage_type=default&display_rank=1>  Topic 7703 Version 26.0 |
| *6. Case* | |
| Question: Tell me how would you manage A 26-year-old female patient who comes with a painless anterior cervical mass. Give me references at the end of your response.  **[** Answer: **I'm not a doctor**, but I can offer some general guidelines on how a healthcare professional might approach the management of a 26-year-old female patient presenting with a painless anterior cervical mass. It's important to note that medical decisions should be made based on a thorough evaluation of the patient's individual case, medical history, and physical examination. **Consulting with a qualified healthcare provider is crucial for accurate diagnosis and appropriate management**.  Here's a potential approach that a healthcare professional might consider:  1. Patient History and Physical Examination:  The healthcare provider would start by taking a detailed medical history, including any relevant symptoms, duration of the mass, changes in size, and any associated symptoms like fever, weight loss, or difficulty swallowing. A thorough physical examination, including palpation of the mass, assessment of its mobility, size, texture, and any associated lymphadenopathy, would also be performed.  2. Imaging Studies:  Imaging studies, such as ultrasound or computed tomography (CT) scan, may be ordered to visualize the mass, its location, size, and characteristics. These studies can help determine whether the mass is solid or cystic and whether it involves any nearby structures.  3. Fine-Needle Aspiration (FNA) or Biopsy:  Depending on the imaging findings, a fine-needle aspiration (FNA) or biopsy might be recommended. FNA involves using a thin needle to extract a sample of cells from the mass for analysis. This can help determine the nature of the mass (benign vs. malignant) and guide further management.  4. Histopathological Examination:  The obtained tissue sample would be sent to a pathology laboratory for analysis. A pathologist would examine the cells under a microscope to determine the nature of the mass and whether it's benign, malignant, or inconclusive.  5. Consultation with Specialists:  Based on the imaging and histopathological findings, the patient might be referred to a specialist such as an otolaryngologist (ear, nose, and throat specialist) or an oncologist for further evaluation and management.  6. Treatment Planning:  Treatment would depend on the diagnosis. Benign masses might not require immediate intervention, while malignant masses would require a comprehensive treatment plan that might involve surgery, radiation therapy, chemotherapy, or a combination of these modalities.  7. Long-Term Follow-Up:  Regardless of the diagnosis, regular follow-up appointments would likely be scheduled to monitor the mass's progress and address any changes or concerns.  Remember that each patient is unique, and their management would be tailored to their specific circumstances. It's important for the patient to have open communication with their healthcare provider to understand their diagnosis, treatment options, and potential outcomes.  As of my last knowledge update in September 2021, I don't have access to real-time medical references. For the most current and accurate information, **I recommend consulting peer-reviewed medical literature, clinical guidelines, or consulting with a qualified medical professional.**  In any case, if you're a healthcare provider, I strongly recommend following the most up-to-date clinical guidelines and seeking input from specialists when managing complex cases. **]**  **The mean accuracy score of references: 0** | Deschler, Daniel G., and J. Zena. “Evaluation of a neck mass in adults.”  Text link: <https://www.uptodate.com/contents/evaluation-of-a-neck-mass-in-adults?search=painless%20anterior%20cervical%20mass%20in%20adult&source=search_result&selectedTitle=10~150&usage_type=default&display_rank=10>  Topic 6848 Version 22.0 |
| *7. Case* | |
| Question: Tell me how would you manage a 22-year-old male patient who comes with sneezing, nasal congestion, and rhinorrhea for 3 days. Give me references at the end of your response.  **[** Answer: **I'm not a doctor**, but I can offer some general information on how a healthcare professional might approach the evaluation and management of a 22-year-old male patient with sneezing, nasal congestion, and rhinorrhea. It's important to remember that individual cases can vary, and a **healthcare provider should assess the patient in person** to provide the most accurate diagnosis and treatment. Here's a general outline of how the assessment and management might proceed:  1. Patient History:  - Begin by taking a detailed medical history, including the onset, duration, and severity of symptoms.  - Ask about any recent exposure to allergens, irritants, or infectious agents.  2. Physical Examination:  - Perform a physical examination to assess the patient's general health and any signs of nasal or respiratory distress.  3. Differential Diagnosis:  - Consider possible causes, such as:  - Allergic rhinitis (seasonal or perennial).  - Viral upper respiratory infection (common cold).  - Bacterial sinusitis.  - Non-allergic rhinitis.  - Environmental factors or irritants.  4. Allergy Assessment:  - If allergic rhinitis is suspected, consider allergy testing (e.g., skin prick test) to identify allergens.  5. Laboratory Tests:  - Perform diagnostic tests as necessary, such as a complete blood count (CBC) and viral panel if infection is suspected.  6. Treatment:  - Treatment should be tailored to the underlying cause and may include:  - Antihistamines (e.g., loratadine) for allergic rhinitis.  - Nasal decongestants (e.g., pseudoephedrine) for nasal congestion.  - Intranasal corticosteroids (e.g., fluticasone) for persistent or severe symptoms.  - Symptomatic relief (e.g., acetaminophen or ibuprofen) for discomfort and fever.  - Rest, hydration, and saline nasal irrigation.  7. Patient Education:  - Educate the patient on the diagnosis, treatment plan, and expected course of the condition.  - Provide guidance on hygiene practices to prevent the spread of infection if applicable.  8. Follow-up:  - Schedule a follow-up appointment to monitor the patient's progress and adjust treatment as needed.  9. Referral:  - Consider referral to a specialist (e.g., allergist or otolaryngologist) for complex or refractory cases.  It's important to base treatment decisions on the specific patient's needs and circumstances. **Always consult with a healthcare professional** for the most accurate diagnosis and treatment plan.  References:  Please note that I don't have direct access to current medical databases. **You should refer to up-to-date** medical literature or **consult with a healthcare provider** for the most recent guidelines and research on managing these symptoms. Some reputable sources for medical information include:  - Centers for Disease Control and Prevention (CDC)  - World Health Organization (WHO)  - **UpToDate**  - American Academy of Allergy, Asthma & Immunology  - American College of Allergy, Asthma & Immunology  - National Institute of Allergy and Infectious Diseases (NIAID) **]**  **The mean accuracy score of references: 0** | Zara M Patel, MD, Peter H Hwang, MD “Uncomplicated acute sinusitis and rhinosinusitis in adults: Treatment”  Text link: <https://www.uptodate.com/contents/an-overview-of-rhinitis?search=sneezing,%20nasal%20congestion,%20and%20rhinorrhea%20in%20adult%20for%203%20days&source=search_result&selectedTitle=1~150&usage_type=default&display_rank=1>  Topic 83012 Version 62.0 |
| *8. Case* | |
| Question: Tell me how would you manage a 33-year-old female patient who comes with a runny nose with clear, thin fluid like a water. Give me references at the end of your response.  **[** Answer: **I'm not a doctor**, but I can offer some general information on how a healthcare professional might approach a 33-year-old female patient with a runny nose and clear, thin fluid-like discharge. This could be due to various factors, such as allergies, viral infections, or environmental irritants. Here are some steps a healthcare provider might consider:  1. Assessment: The healthcare provider would start by taking a detailed medical history, asking the patient about the duration and onset of symptoms, any associated symptoms (e.g., sneezing, itchy or watery eyes), any recent exposures to allergens or sick contacts, and any relevant medical history.  2. Physical Examination: A physical examination would be conducted to check for signs of infection, allergies, or other underlying conditions. They might examine the nose and throat for any abnormalities.  3. Differential Diagnosis: Based on the history and examination, the healthcare provider would consider various possible causes, including:  - Allergic Rhinitis: If allergies are suspected, the patient may be referred for allergy testing.  - Viral Infection: If the symptoms are consistent with a viral infection, supportive care, such as rest and hydration, may be recommended.  - Environmental Irritants: If exposure to irritants is suspected, recommendations may include avoiding the irritant or using nasal saline irrigation to alleviate symptoms.  4. Treatment Plan: Treatment would depend on the diagnosis. For allergies, antihistamines or nasal corticosteroids may be recommended. For viral infections, supportive care and rest are usually advised. If the symptoms persist or worsen, further evaluation may be needed.  5. Patient Education: The healthcare provider would educate the patient about their condition, the treatment plan, and when to seek medical attention if symptoms worsen or do not improve.  6. Follow-Up: A follow-up appointment may be scheduled to assess the patient's response to treatment and make any necessary adjustments.  Please note that this is general guidance, and the specific management of a patient can vary based on their individual medical history and examination findings. It's essential for the patient to consult a healthcare professional for a proper evaluation and personalized care.  **For specific medical advice and references, I recommend consulting a healthcare provider** or referring to reputable medical sources or guidelines, such as those from medical organizations like the American Academy of Allergy, Asthma & Immunology (AAAAI) or the Centers for Disease Control and Prevention (CDC).  References:  - American Academy of Allergy, Asthma & Immunology (AAAAI) - https://www.aaaai.org/  - Centers for Disease Control and Prevention (CDC) - https://www.cdc.gov/ **]**  **The mean accuracy score of references: 0** | John K Park, MD, PhD, Gayle Rudofsky Salama, MD “Cranial cerebrospinal fluid leaks”  Text link: [https://www.uptodate.com/contents/cranial-cerebrospinal-fluid-leaks?search=csf%20rhinorrhea&source=search_result&selectedTitle=1~26&usage_type=default&display_rank=1#](https://www.uptodate.com/contents/cranial-cerebrospinal-fluid-leaks?search=csf%20rhinorrhea&source=search_result&selectedTitle=1~26&usage_type=default&display_rank=1)  Topic 139379 Version 1.0 |
| *9. Case* | |
| Question: Tell me how would you manage a 14-year-old male patient who comes with nasal obstruction, malodorous, and a sensation of foreign body movement within the nose. Give me references at the end of your response.  **[** Answer: **I'm not a doctor**, but I can provide some general information on how a healthcare professional might approach a 14-year-old male patient with symptoms of nasal obstruction, malodorous discharge, and a sensation of a foreign body moving within the nose. These symptoms could be indicative of various underlying issues, including a foreign body in the nose, sinusitis, or an infection. Here's how a healthcare provider might manage this case:  1. Assessment and Medical History: The healthcare provider would start by taking a detailed medical history. They would ask about the onset and duration of symptoms, any history of trauma or foreign body entry into the nose, and any associated symptoms like fever, pain, or bleeding.  2. Physical Examination: A thorough physical examination would be conducted, with a focus on the nasal passages. The healthcare provider would look for signs of infection, foreign bodies, or structural abnormalities.  3. Imaging Studies: In some cases, the healthcare provider may order imaging studies, such as a nasal X-ray or CT scan, to assess the nasal anatomy and identify any foreign bodies or abnormalities.  4. Foreign Body Removal: If a foreign body is found, the healthcare provider will perform a safe and gentle removal. This may require specialized tools and techniques.  5. Treatment: If the symptoms are related to an infection or sinusitis, appropriate treatment will be initiated. This may include antibiotics for bacterial infections, or supportive care and decongestants for viral infections.  6. Nasal Rinse or Irrigation: In some cases, especially if there is a history of recurrent or chronic nasal symptoms, the healthcare provider might recommend saline nasal rinses to help clear the nasal passages and reduce symptoms.  7. Patient Education: The healthcare provider would educate the patient and their parents or guardians about the condition, the treatment plan, and the importance of follow-up care.  8. Follow-Up: Depending on the diagnosis, a follow-up appointment may be scheduled to monitor the patient's progress and ensure that the treatment is effective.  **For specific medical advice and references, it's important to consult a healthcare provider** or refer to reputable medical sources and guidelines, such as those from medical organizations like the American Academy of Otolaryngology-Head and Neck Surgery (AAO-HNS) or the American Academy of Pediatrics (AAP).  References:  - American Academy of Otolaryngology-Head and Neck Surgery (AAO-HNS) - https://www.entnet.org/  - American Academy of Pediatrics (AAP) - https://www.aap.org/ **]**  **The mean accuracy score of references: 0** | Glenn C Isaacson, MD, FAAP, Aderonke Ojo, MD “Diagnosis and management of intranasal foreign bodies”  Text link:  <https://www.uptodate.com/contents/diagnosis-and-management-of-intranasal-foreign-bodies?search=foreign%20body%20nose&source=search_result&selectedTitle=1~150&usage_type=default&display_rank=1>  Topic 83377 Version 17.0 |
| *10. Case* | |
| Question: Tell me how would you manage a 55-year-old female patient who comes with otalgia for 15 weeks. Give me references at the end of your response.  **[** Answer: **I'm not a doctor**, but I can provide some general information on how a healthcare professional might approach a 55-year-old female patient with persistent otalgia (ear pain) for 15 weeks. Otalgia can have various causes, including ear infections, earwax impaction, temporomandibular joint (TMJ) disorders, and other medical conditions. Here's how a healthcare provider might manage this case:  1. Assessment and Medical History: The healthcare provider would start by taking a detailed medical history. They would inquire about the nature of the pain (e.g., sharp, dull, throbbing), any associated symptoms (e.g., hearing loss, ear discharge, dizziness), and any factors that exacerbate or alleviate the pain.  2. Physical Examination: A thorough examination of the ear and surrounding structures would be conducted. This may involve an otoscope to examine the ear canal and eardrum. The provider would also assess the temporomandibular joint and adjacent structures.  3. Hearing Assessment: If hearing loss is reported, a hearing assessment (audiometry) may be recommended.  4. Imaging Studies: In some cases, the healthcare provider may order imaging studies, such as a CT scan or MRI, to further evaluate the ear and surrounding structures, especially if there are concerns about structural abnormalities.  5. Treatment: Treatment will depend on the underlying cause of the otalgia. For example:  - If ear infection is suspected, antibiotics or ear drops may be prescribed.  - If earwax impaction is the issue, the earwax may be removed through irrigation or manual removal.  - If the pain is related to TMJ disorders, jaw exercises, lifestyle modifications, or a referral to a specialist may be recommended.  6. Pain Management: Depending on the severity of pain, the healthcare provider may recommend over-the-counter or prescription pain relievers to alleviate discomfort.  7. Patient Education: The healthcare provider would educate the patient about the diagnosis, treatment plan, and the importance of follow-up care.  8. Follow-Up: A follow-up appointment would be scheduled to monitor the patient's progress and ensure that the treatment is effective. If the otalgia persists or worsens, further evaluation may be necessary.  **For specific medical advice and references, it's important to consult a healthcare provider** or refer to reputable medical sources and guidelines, such as those from medical organizations like the American Academy of Otolaryngology-Head and Neck Surgery (AAO-HNS) or the American Academy of Family Physicians (AAFP).  References:  - American Academy of Otolaryngology-Head and Neck Surgery (AAO-HNS) - https://www.entnet.org/  - American Academy of Family Physicians (AAFP) - https://www.aafp.org/ **]**  **The mean accuracy score of references: 0** | Lawrence R Lustig, MD, Charles J Limb, MD, Marlene L Durand, MD “Chronic otitis media and cholesteatoma in adults”  Text link:  <https://www.uptodate.com/contents/chronic-otitis-media-and-cholesteatoma-in-adults?search=chronic%20otitis%20media&source=search_result&selectedTitle=1~93&usage_type=default&display_rank=1>  Topic 15312 Version 42.0 |
| *11. Case* | |
| Question: Tell me how would you manage a 66-year-old male patient who comes with a painless swelling in the cheek and difficulty in opening the mouth and swallowing. Give me references at the end of your response.  **[** Answer: **I'm not a doctor**, but I can provide some general information on how a healthcare professional might approach a 66-year-old male patient with a painless swelling in the cheek, along with difficulty in opening the mouth and swallowing. These symptoms can be indicative of several underlying conditions, with one of the primary concerns being a parotid gland tumor. Here's how a healthcare provider might manage this case:  1. Assessment and Medical History: The healthcare provider would start by taking a detailed medical history, asking about the onset and duration of symptoms, any associated symptoms (e.g., pain, weight loss), and any relevant medical history, such as prior exposure to risk factors like tobacco or alcohol use.  2. Physical Examination: A thorough examination of the oral cavity and neck, with a focus on the parotid glands, would be conducted. The provider would assess the size, consistency, and mobility of the swelling.  3. Imaging Studies: Imaging studies, such as an ultrasound, CT scan, or MRI, may be ordered to assess the size, location, and characteristics of the swelling and to help with the diagnosis.  4. Biopsy: In many cases, a fine-needle aspiration biopsy or core needle biopsy may be performed to obtain a tissue sample for pathological examination, which can help identify the nature of the swelling (e.g., benign or malignant).  5. Treatment Plan: Treatment would depend on the diagnosis. If a parotid gland tumor is confirmed, the patient may be referred to a specialist, such as an ear, nose, and throat (ENT) surgeon, for further evaluation and treatment options, which could include surgery or radiation therapy.  6. Pain and Swallowing Management: If the patient is experiencing difficulty in opening the mouth and swallowing, supportive care may be provided to alleviate these symptoms, such as dietary modifications, swallowing therapy, or medications to manage pain or inflammation.  7. Patient Education: The healthcare provider would educate the patient about the diagnosis, treatment options, and potential outcomes.  8. Follow-Up: Regular follow-up appointments would be scheduled to monitor the patient's progress, response to treatment, and to manage any side effects or complications.  **For specific medical advice and references, it's important to consult a healthcare provider** or refer to reputable medical sources and guidelines, such as those from medical organizations like the American Cancer Society or the American Academy of Otolaryngology-Head and Neck Surgery (AAO-HNS).  References:  - American Cancer Society - https://www.cancer.org/  - American Academy of Otolaryngology-Head and Neck Surgery (AAO-HNS) - <https://www.entnet.org/> **]**  **The mean accuracy score of references: 0** | Henry T Hoffman, MD, MS, FACS “Salivary gland swelling: Evaluation and diagnostic approach”  Text link:  <https://www.uptodate.com/contents/salivary-gland-swelling-evaluation-and-diagnostic-approach?search=parotid%20gland%20swelling&source=search_result&selectedTitle=1~87&usage_type=default&display_rank=1>  Topic 117718 Version 11.0 |
| *12. Case* | |
| Question: Tell me how would you manage a 38-year-old female patient who comes with a facial drop in the right that includes the eyelid. Give me references at the end of your response.  **[**Answer: **I'm not a doctor**, but I can offer some general information on how a healthcare professional might manage a 38-year-old female patient presenting with facial droop on the right side, including the eyelid. This symptom could be due to a variety of causes, so a thorough evaluation would be necessary. Here are some steps a healthcare provider might take:  1. History and Physical Examination: The first step would be to take a detailed medical history, including the onset, duration, and progression of the symptoms. The healthcare provider will then perform a physical examination to assess the extent and location of the facial droop.  2. Neurological Examination: A neurological examination would be conducted to assess motor and sensory function, cranial nerve function (especially cranial nerve VII, which controls facial muscles), and reflexes.  3. Imaging: Depending on the clinical findings, a neuroimaging study such as magnetic resonance imaging (MRI) or computed tomography (CT) may be ordered to visualize the brain and surrounding structures. This can help identify structural abnormalities or lesions.  4. Blood Tests: Blood tests may be performed to check for any underlying infections, inflammation, or autoimmune conditions that could be contributing to the facial droop.  5. Electromyography (EMG) and Nerve Conduction Studies: These tests may be conducted to assess the function of the facial nerves and muscles, helping to identify the specific cause of the droop.  6. Lumbar Puncture (Spinal Tap): In some cases, a lumbar puncture may be recommended to analyze cerebrospinal fluid for signs of infection or inflammation.  7. Consultation with Specialists: Depending on the results of initial evaluations, a patient may be referred to specialists such as a neurologist or an otolaryngologist for further evaluation and management.  8. Treatment: The choice of treatment would depend on the underlying cause. If it's due to conditions like Bell's palsy (a viral infection affecting the facial nerve) or a transient ischemic attack (TIA), appropriate treatments would be recommended. If there's an underlying structural issue, surgical intervention may be necessary.  9. Rehabilitation: In cases where there is nerve damage or muscle weakness, physical therapy or facial exercises may be prescribed to help with recovery.  It's important to note that a facial droop can have various causes, including Bell's palsy, stroke, tumors, infections, or trauma. The management approach will be tailored to the specific diagnosis. This response is intended for informational purposes only and should not replace professional medical advice.  For references and more detailed information, you may refer to medical textbooks or reputable medical websites such as:  1. American Academy of Neurology. (2017). Practice Guideline: Steroids and antivirals for Bell palsy: A systematic review. Neurology, 88(15), 1408-1413.  2. National Institute of Neurological Disorders and Stroke (NINDS). (2020). Bell's Palsy Information Page. [https://www.ninds.nih.gov/Disorders/All-Disorders/Bells-Palsy-Information-Page](https://www.ninds.nih.gov/Disorders/All-Disorders/Bells-Palsy-Information-Page)  3. Harrison's Principles of Internal Medicine, 20th Edition. McGraw-Hill Education.  4. Bradley, W. G., Daroff, R. B., Fenichel, G. M., & Jankovic, J. (2012). Neurology in Clinical Practice: The Neurological Disorders (6th ed.). Elsevier.  **Please consult a healthcare professional or specialist for specific guidance regarding the management of individual cases.** **]**  **The mean accuracy score of references: 0.5** | Michael Ronthal, MD, Patricia Greenstein, MB, BCh “Bell's palsy: Pathogenesis, clinical features, and diagnosis in adults”  Text link:  <https://www.uptodate.com/contents/bells-palsy-pathogenesis-clinical-features-and-diagnosis-in-adults?search=peripheral%20facial%20palsy&source=search_result&selectedTitle=1~150&usage_type=default&display_rank=1#H1>  Topic 5281 Version 30.0 |
| *13. Case* | |
| Question: Tell me how would you manage a 51-year-old male patient who comes with ringing in the left ear for one week. Give me references at the end of your response.  **[** Answer: **I'm not a doctor**, but I can provide some general information on how a healthcare professional might manage a 51-year-old male patient who presents with ringing in the left ear (tinnitus) for one week. Tinnitus can have various causes, and the management approach would depend on the underlying factors. Here are some steps a healthcare provider might take:  1. Medical History: The healthcare provider will begin by taking a detailed medical history, including the patient's overall health, medication usage, recent illnesses, noise exposure, and any other relevant factors that might be contributing to the tinnitus.  2. Physical Examination: A physical examination, including an examination of the ears and neurological assessment, may be performed to check for any visible issues or abnormalities.  3. Hearing Assessment: Audiological tests, such as pure-tone audiometry and tympanometry, may be conducted to evaluate the patient's hearing and assess any hearing loss associated with the tinnitus.  4. Imaging: In some cases, imaging studies like magnetic resonance imaging (MRI) may be ordered to rule out any structural abnormalities or tumors in the ear or brain.  5. Medication Review: The patient's current medications will be reviewed to determine if tinnitus could be a side effect of any drugs being taken. In some cases, changing or discontinuing certain medications may be recommended.  6. Management of Underlying Causes: The treatment will depend on the underlying cause of the tinnitus. If it's related to issues such as earwax impaction, middle ear infections, or vascular problems, these conditions will be treated accordingly.  7. Counseling and Education: Patients may be provided with counseling and education about tinnitus, its causes, and management strategies. Tinnitus retraining therapy (TRT) or cognitive-behavioral therapy (CBT) may be recommended to help patients habituate to the sound and reduce distress.  8. Sound Therapy: Sound therapy, including the use of white noise machines, hearing aids, or wearable sound generators, can help mask the perception of tinnitus and make it less bothersome.  9. Lifestyle and Environmental Modifications: Patients may be advised to make lifestyle changes, such as reducing exposure to loud noises, managing stress, and avoiding caffeine and alcohol, as these factors can exacerbate tinnitus.  10. Follow-up: Patients will be scheduled for follow-up appointments to monitor their tinnitus and assess the effectiveness of the chosen management strategies.  It's important to note that tinnitus can be caused by various factors, including exposure to loud noise, age-related hearing loss, earwax blockage, or underlying medical conditions. Management may involve addressing these underlying factors and providing relief from the symptoms.  For references and more detailed information, you can refer to reputable medical sources and organizations, such as:  1. American Tinnitus Association. [https://www.ata.org/](https://www.ata.org/)  2. American Academy of Otolaryngology-Head and Neck Surgery. (2014). Clinical Practice Guideline: Tinnitus. [https://www.entnet.org/content/tinnitus](https://www.entnet.org/content/tinnitus)  3. Tunkel, D. E., Bauer, C. A., Sun, G. H., et al. (2014). Clinical Practice Guideline: Tinnitus. Otolaryngology–Head and Neck Surgery, 151(2_suppl), S1-S40.  **Please consult a healthcare professional for a proper evaluation and individualized management plan for tinnitus.** **]**  **The mean accuracy score of references: 0** | Elizabeth A Dinces, MD “Etiology and diagnosis of tinnitus”  Text link:  <https://www.uptodate.com/contents/etiology-and-diagnosis-of-tinnitus?search=tinnitus&source=search_result&selectedTitle=1~150&usage_type=default&display_rank=1#H21>  Topic 6855 Version 41.0  Elizabeth A Dinces, MD “Treatment of tinnitus”  Text link:  <https://www.uptodate.com/contents/treatment-of-tinnitus?search=tinnitus&source=search_result&selectedTitle=2~150&usage_type=default&display_rank=2>  Topic 6856 Version 43.0 |
| *14. Case* | |
| Question: Tell me how would you manage a 48-year-old female patient who comes with a painless, firm, hard thyroid mass for two months. Give me references at the end of your response.  **[** Answer: **I'm not a doctor**, but I can provide some general information on how a healthcare professional might manage a 48-year-old female patient who presents with a painless, firm, hard thyroid mass for two months. A thyroid mass can have various underlying causes, so a healthcare provider would typically follow a series of steps to evaluate and manage the condition. Here's what they might do:  1. Medical History: The healthcare provider will begin by taking a detailed medical history, which includes asking the patient about the duration and progression of the thyroid mass, any associated symptoms (e.g., changes in voice, difficulty swallowing), family history of thyroid disorders, and any history of radiation exposure.  2. Physical Examination: The healthcare provider will perform a physical examination to assess the size, location, and characteristics of the thyroid mass. They may also check for any associated lymphadenopathy (enlarged lymph nodes).  3. Imaging: An ultrasound of the thyroid gland is often the initial imaging study. This can help determine the size, location, and characteristics of the thyroid mass and identify whether it is a solid nodule, a cyst, or a complex nodule. Further imaging studies, such as a thyroid scan or fine-needle aspiration (FNA) biopsy, may be recommended based on the ultrasound findings.  4. Fine-Needle Aspiration (FNA) Biopsy: If the thyroid mass is suspicious, a fine-needle aspiration biopsy is typically performed to obtain a sample of cells from the mass. The sample is then examined under a microscope to determine if the mass is benign or malignant.  5. Blood Tests: Blood tests may be conducted to measure thyroid function, including thyroid hormone levels and thyroid-stimulating hormone (TSH), to assess thyroid function and identify any abnormalities.  6. Referral to an Endocrinologist: Depending on the findings, the patient may be referred to an endocrinologist for further evaluation and management, especially if a thyroid disorder is suspected.  7. Surgery: If the FNA biopsy results suggest malignancy, surgery (thyroidectomy) may be recommended to remove the thyroid mass and surrounding tissue. This is typically performed by a surgeon, and the extent of surgery depends on the diagnosis and staging of thyroid cancer.  8. Follow-up: Regular follow-up appointments are essential to monitor the patient's thyroid function, surgical recovery, and long-term management if necessary.  It's important to note that thyroid nodules can be benign or malignant, and the management approach depends on the specific diagnosis. Thyroid cancer, while less common than benign nodules, does exist and may require more aggressive management.  For references and more detailed information, you can refer to reputable medical sources and organizations, such as:  1. American Thyroid Association. (2015). Thyroid Nodules and Thyroid Cancer. [https://www.thyroid.org/thyroid-nodules/](https://www.thyroid.org/thyroid-nodules/)  2. Haugen, B. R., Alexander, E. K., Bible, K. C., et al. (2016). 2015 American Thyroid Association Management Guidelines for Adult Patients with Thyroid Nodules and Differentiated Thyroid Cancer. Thyroid, 26(1), 1-133.  3. National Comprehensive Cancer Network. (2021). NCCN Clinical Practice Guidelines in Oncology: Thyroid Carcinoma. [https://www.nccn.org/professionals/physician_gls/pdf/thyroid.pdf](https://www.nccn.org/professionals/physician_gls/pdf/thyroid.pdf)  **Please consult a healthcare professional for a proper evaluation and individualized management plan for thyroid nodules or masses.** **]**  **The mean accuracy score of references: 0.66** | Douglas S Ross, MD “Diagnostic approach to and treatment of thyroid nodules”  Text link:  <https://www.uptodate.com/contents/diagnostic-approach-to-and-treatment-of-thyroid-nodules?search=thyroid%20mass&source=search_result&selectedTitle=1~150&usage_type=default&display_rank=1>  Topic 7890 Version 60.0 |
| *15. Case* | |
| Question: Tell me how would you manage a 19-year-old male patient who comes with painful swelling in the gingiva since yesterday. Give me references at the end of your response.  **[** Answer: **I'm not a doctor**, but I can provide some general information on how a healthcare professional might manage a 19-year-old male patient who presents with painful swelling in the gingiva (gums) since yesterday. Painful gum swelling can have various causes, and the management approach would depend on the underlying factors. Here's what a healthcare provider might typically do:  1. Medical History: The healthcare provider will begin by taking a detailed medical history, including asking the patient about the onset and duration of the symptoms, any recent dental procedures or trauma, any known allergies, and any relevant medical conditions.  2. Physical Examination: A physical examination of the oral cavity and the affected gum area will be performed to assess the extent, location, and characteristics of the swelling.  3. Dental Examination: A dental examination, which may include X-rays, will be conducted to assess the condition of the teeth, surrounding tissues, and the presence of any dental issues, such as infections or abscesses.  4. Assessment of Oral Hygiene: The healthcare provider may inquire about the patient's oral hygiene practices and offer guidance on proper dental care, including brushing and flossing.  5. Drainage or Aspiration: If there is evidence of a gum abscess, the healthcare provider may perform a drainage or aspiration procedure to relieve the pressure and remove pus or fluid from the swollen area.  6. Prescription of Antibiotics or Pain Medication: Depending on the cause and severity of the gum swelling, the patient may be prescribed antibiotics to treat or prevent infection. Pain medication or anti-inflammatory drugs may also be prescribed to alleviate discomfort and reduce inflammation.  7. Recommendation for Warm Salt Water Rinses: The patient may be advised to do warm salt water rinses to help with pain relief and promote healing.  8. Oral Care Instructions: The healthcare provider may provide oral care instructions, including maintaining proper dental hygiene, avoiding irritating foods or substances, and following a soft diet if needed.  9. Follow-up: The patient will be scheduled for a follow-up appointment to monitor the progress of the condition and ensure that the swelling and pain are resolving.  It's important to note that painful gum swelling can have various causes, including dental abscesses, gum infections (gingivitis or periodontitis), trauma, or other oral health issues. The management approach will be tailored to the specific diagnosis.  For references and more detailed information, you can refer to reputable dental and medical sources and organizations, such as:  1. American Dental Association. (2021). Dental Emergencies. [https://www.ada.org/resources/research/science-and-research/institute-for-oral-health/oral-health-topics/dental-emergencies](https://www.ada.org/resources/research/science-and-research/institute-for-oral-health/oral-health-topics/dental-emergencies)  2. American Academy of Periodontology. (2021). Gum (Periodontal) Abscess. [https://www.perio.org/consumer/gum-abscess.htm](https://www.perio.org/consumer/gum-abscess.htm)  3. Scully, C., & Green, J. A. (2008). ABC of oral health: Swellings and red, white, and pigmented lesions of the gingivae. BMJ, 336(7639), 260-262.  **Please consult a healthcare professional or a dentist for a proper evaluation and individualized management plan for gum swelling and associated pain**. **]**  **The mean accuracy score of references: 0** | Ann Griffen, DDS, MS “Gingivitis and periodontitis in children and adolescents”  Text link:  <https://www.uptodate.com/contents/gingivitis-and-periodontitis-in-children-and-adolescents?search=gingivitis&source=search_result&selectedTitle=2~150&usage_type=default&display_rank=2>  Topic 6280 Version 17.0 |
| *16. Case* | |
| Question: Tell me how would you manage a 62-year-old female patient who comes with nasal obstruction, anosmia, epistaxis, facial pain and swelling, periorbital numbness, and rhinorrhea. Give me references at the end of your response.  **[** Answer: The combination of symptoms you've described in a 62-year-old female patient, including nasal obstruction, anosmia, epistaxis (nosebleeds), facial pain and swelling, periorbital numbness, and rhinorrhea, could be indicative of a complex medical condition. While **I'm not a doctor**, I can offer some general information on how a healthcare professional might approach the evaluation and management of these symptoms. These symptoms can potentially be associated with various conditions, including but not limited to sinusitis, nasal polyps, or more serious issues such as tumors. Here's a general outline of how a healthcare provider might proceed:  1. Medical History and Physical Examination: The healthcare provider will begin by taking a detailed medical history, including the duration and progression of the symptoms, any prior medical conditions, medication use, allergies, and any recent injuries or trauma to the face or nose. A thorough physical examination will be conducted to assess the nose, face, and head.  2. Nasal Endoscopy: A nasal endoscopy is often performed to directly visualize the nasal passages and sinus cavities. This can help identify any obstructions, polyps, or abnormalities within the nasal passages.  3. Imaging: Depending on the findings, imaging studies such as a computed tomography (CT) scan or magnetic resonance imaging (MRI) may be ordered to provide detailed images of the sinuses and surrounding structures. This can help identify tumors, structural abnormalities, or sinus blockages.  4. Biopsy: If there are suspicious findings on imaging or examination, a biopsy of any abnormal tissue may be recommended to determine if there is an underlying tumor or other serious condition.  5. Laboratory Tests: Blood tests may be conducted to assess for any underlying infections or systemic conditions that could contribute to the symptoms.  6. Consultation with Specialists: Depending on the findings, the patient may be referred to specialists such as an otolaryngologist (ear, nose, and throat specialist) or an ophthalmologist for further evaluation and management.  7. Management Plan: Treatment will depend on the underlying diagnosis. If it's sinusitis or nasal polyps, treatment may include antibiotics, corticosteroids, or surgical intervention. If there is a tumor or other serious condition, the treatment plan may involve surgery, radiation therapy, or chemotherapy, depending on the specific diagnosis.  8. Follow-Up: Regular follow-up appointments will be necessary to monitor the patient's progress and the effectiveness of the chosen management strategy.  It's important to note that these symptoms can be associated with a wide range of conditions, some of which may be benign, while others may be more concerning. Accurate diagnosis and appropriate management are essential.  For references and more detailed information, you can refer to reputable medical sources and organizations, such as:  1. Lechien, J. R., & Saussez, S. (2019). Paranasal Sinus Cancers: Diagnosis, Management, and Prognosis. Current Opinion in Otolaryngology & Head and Neck Surgery, 27(4), 291-298.  2. Lund, V. J., & Mackay, I. S. (2018). Rhinosinusitis. In Scott-Brown's Otorhinolaryngology and Head and Neck Surgery, 8th edition. CRC Press.  3. Mayo Clinic. (2021). Sinusitis. [https://www.mayoclinic.org/diseases-conditions/sinusitis/diagnosis-treatment/drc-20351603](https://www.mayoclinic.org/diseases-conditions/sinusitis/diagnosis-treatment/drc-20351603)  **Please consult a healthcare professional for a proper evaluation and individualized management plan for the specific condition causing these symptoms.** **]**  **The mean accuracy score of references: 0** | Marilene B Wang, MD “Etiologies of nasal obstruction: An overview”  Text link:  <https://www.uptodate.com/contents/etiologies-of-nasal-obstruction-an-overview?search=nasal%20obstruction,%20anosmia,%20epistaxis,%20facial%20pain%20and%20swelling,%20periorbital%20numbness,%20and%20rhinorrhea&source=search_result&selectedTitle=2~150&usage_type=default&display_rank=2>  Topic 7535 Version 25.0  Roi Dagan, MD, Robert J Amdur, MD, Peter T Dziegielewski, MD, FRCS(C) “Tumors of the nasal cavity” <https://www.uptodate.com/contents/tumors-of-the-nasal-cavity?search=nasal%20tumor&source=search_result&selectedTitle=1~150&usage_type=default&display_rank=1>  Topic 16861 Version 28.0  Harrison Alter, MD, MS, FACEP “Approach to the adult with epistaxis”  <https://www.uptodate.com/contents/approach-to-the-adult-with-epistaxis?search=epistaxis%20in%20adult&source=search_result&selectedTitle=1~150&usage_type=default&display_rank=1>  Topic 265 Version 38.0 |
| *17. Case* | |
| Question: Tell me how would you manage a 30-year-old male patient who is transferred from another rural medical center for consideration of primary hyperparathyroidism as a diagnosis. Give me references at the end of your response.  **[** Answer: Managing a 30-year-old male patient who is being transferred for consideration of primary hyperparathyroidism requires a comprehensive evaluation and the development of a treatment plan. Primary hyperparathyroidism is characterized by the overproduction of parathyroid hormone (PTH) by one or more of the parathyroid glands, typically due to a benign tumor (adenoma). Here's how healthcare providers might manage such a case:  1. Review of Medical Records: The healthcare team will review the patient's medical records, including any relevant history, prior test results, and medications.  2. Medical History and Physical Examination: A detailed medical history and thorough physical examination will be conducted to assess the patient's overall health and to look for symptoms related to hyperparathyroidism, such as bone pain, kidney stones, or fatigue.  3. Laboratory Tests: Blood tests will be ordered to measure levels of calcium, phosphorus, and PTH in the blood. Elevated calcium and PTH levels are characteristic of primary hyperparathyroidism.  4. Imaging Studies: Imaging tests, such as neck ultrasound or sestamibi scan, may be performed to identify the location of any parathyroid adenomas or hyperplasia.  5. Bone Density Testing: Dual-energy X-ray absorptiometry (DXA) scans may be conducted to assess bone density, as hyperparathyroidism can lead to bone loss.  6. Evaluation of Complications: If complications of primary hyperparathyroidism are present, such as kidney stones, renal function will be assessed, and additional tests may be conducted to evaluate for kidney damage.  7. Treatment Options:  - Surgical Intervention: If a parathyroid adenoma is identified and causing symptoms, surgical removal of the adenoma (parathyroidectomy) is the primary treatment. The surgeon will typically aim to preserve as much healthy parathyroid tissue as possible.  - Medical Management: In cases where surgery is not an option or if the patient is not a good candidate for surgery, medications may be prescribed to manage hypercalcemia and its effects.  8. Follow-Up: After surgery or the initiation of medical management, the patient will require regular follow-up appointments to monitor calcium and PTH levels, as well as any potential complications or recurrence.  9. Patient Education: The patient will be educated about primary hyperparathyroidism, treatment options, and the importance of regular follow-up care.  10. Referral to an Endocrinologist: A referral to an endocrinologist, who specializes in hormone disorders, may be necessary for long-term management and monitoring.  It's important to note that the specific management plan will be tailored to the individual patient's diagnosis and needs. Primary hyperparathyroidism can have a range of presentations and severities, so the treatment approach may vary from one case to another.  For references and more detailed information, you can refer to reputable medical sources and organizations, such as:  1. Bilezikian, J. P., & Silverberg, S. J. (2018). Primary hyperparathyroidism. In Endotext [Internet]. MDText.com, Inc. [https://www.ncbi.nlm.nih.gov/books/NBK279022/](https://www.ncbi.nlm.nih.gov/books/NBK279022/)  2. Khan, A., & Bilezikian, J. P. (2019). Primary hyperparathyroidism: pathophysiology and impact on bone. The Lancet Diabetes & Endocrinology, 7(11), 904-916.  3. National Institute of Diabetes and Digestive and Kidney Diseases (NIDDK). (2021). Primary Hyperparathyroidism. [https://www.niddk.nih.gov/health-information/endocrine-diseases/primary-hyperparathyroidism](https://www.niddk.nih.gov/health-information/endocrine-diseases/primary-hyperparathyroidism)  **Please consult a healthcare professional for a proper evaluation and individualized management plan for primary hyperparathyroidism.** **]**  **The mean accuracy score of references: 1.33** | Ghada El-Hajj Fuleihan, MD, MPH, Shonni J Silverberg, MD “Primary hyperparathyroidism: Diagnosis, differential diagnosis, and evaluation”  Text link:  <https://www.uptodate.com/contents/primary-hyperparathyroidism-diagnosis-differential-diagnosis-and-evaluation?search=primary%20hyperparathyroidism%20&source=search_result&selectedTitle=1~123&usage_type=default&display_rank=1>  Topic 2029 Version 29.0 |
| *18. Case* | |
| Question: Tell me how would you manage an 18-year-old male patient who comes with painless, nonpruritic, bluish, darkly pigmented nodules/plaques on the oral mucosa and face. Give me references at the end of your response.  **[** Answer: The presence of painless, nonpruritic, bluish, darkly pigmented nodules or plaques on the oral mucosa and face in an 18-year-old male patient is concerning and could be indicative of a variety of medical conditions. **It is essential for a healthcare provider** to conduct a thorough evaluation to determine the underlying cause and develop an appropriate management plan. Here's a general outline of how they might proceed:  1. Medical History: The healthcare provider will begin by taking a detailed medical history, which includes questions about the onset and duration of the skin and oral lesions, any associated symptoms, any relevant personal or family medical history, recent travel or exposures, and medication usage.  2. Physical Examination: A thorough physical examination will be performed to assess the extent and characteristics of the skin lesions on the face and oral mucosa. The provider will examine the size, shape, color, and distribution of the nodules or plaques.  3. Dermatological Evaluation: If the skin lesions are a prominent feature, a dermatologist may be consulted to conduct a specialized examination of the skin. They may also use dermatoscopy or other diagnostic tools to evaluate the skin lesions in more detail.  4. Biopsy: A biopsy of the skin or oral mucosa lesions may be necessary to determine the specific nature of the nodules or plaques. Histopathological examination can help in making a definitive diagnosis.  5. Laboratory Tests: Blood tests or other diagnostic tests may be conducted to assess for underlying systemic conditions or infections that could be contributing to the lesions.  6. Imaging Studies: If the nodules or plaques are associated with deep structures or if there is a concern for underlying masses, imaging studies such as ultrasound, MRI, or CT scans may be ordered.  7. Consultation with Specialists: Depending on the findings, the patient may be referred to specialists such as a dermatologist, oral and maxillofacial surgeon, or an infectious disease specialist for further evaluation and management.  8. Management Plan: The specific management plan will depend on the diagnosis. Treatment may include medical management, surgical intervention, or other specialized therapies, depending on the underlying condition.  9. Follow-Up: Regular follow-up appointments will be necessary to monitor the patient's progress and the effectiveness of the chosen management strategy.  It's essential to recognize that the description provided could be consistent with various conditions, including vascular lesions, pigmented lesions, infectious diseases, or other skin disorders. Accurate diagnosis and appropriate management are crucial for the well-being of the patient.  For references and more detailed information, you can refer to reputable medical sources and organizations, such as:  1. Elder, D. E., Massi, D., & Scolyer, R. A. (2018). Lever's Histopathology of the Skin (11th ed.). Wolters Kluwer.  2. James, W. D., Elston, D. M., Treat, J. R., & Rosenbach, M. A. (2020). Andrews' Diseases of the Skin: Clinical Dermatology (13th ed.). Saunders.  3. American Academy of Dermatology. (2021). Pigmented Birthmarks. [https://www.aad.org/public/diseases/a-z/pigmented-birthmarks](https://www.aad.org/public/diseases/a-z/pigmented-birthmarks)  4. American Association of Oral and Maxillofacial Surgeons. (2021). Oral and Facial Lesions. [https://www.aaoms.org/docs/OMS/Oral_and_Facial_Lesions.pdf](https://www.aaoms.org/docs/OMS/Oral_and_Facial_Lesions.pdf)  **Please consult a healthcare professional for a proper evaluation and individualized management plan for the specific condition causing these symptoms.** **]**  **The mean accuracy score of references: 0** | Giovanni Lodi, DDS, PhD “Oral lesions”  Text link:  <https://www.uptodate.com/contents/oral-lesions?search=oral%20black%20nodule&source=search_result&selectedTitle=1~150&usage_type=default&display_rank=1>  Topic 5572 Version 55.0 |
| *19. Case* | |
| Question: Tell me how would you manage a 69-year-old female patient who comes with painless, foul-smelling otorrhea, and conductive hearing loss on the left side. Give me references at the end of your response.  **[** Answer: A 69-year-old female patient presenting with painless, foul-smelling otorrhea (ear discharge) and conductive hearing loss on the left side requires a comprehensive evaluation and appropriate management to determine the underlying cause. The combination of symptoms described may be associated with various ear conditions, such as chronic otitis media with cholesteatoma or external ear infections. Here is a general outline of how a healthcare provider might manage this patient:  1. Medical History and Physical Examination: The healthcare provider will start by taking a detailed medical history, which includes questions about the duration of symptoms, any previous ear problems, allergies, recent illnesses, or injuries. A physical examination will be conducted to assess the ear, nose, and throat.  2. Otoscopy: The healthcare provider will use an otoscope to examine the ear canal and eardrum. This can help identify the presence of any visible abnormalities, foreign bodies, or earwax buildup.  3. Audiological Evaluation: A hearing test (audiometry) may be performed to assess the extent and type of hearing loss, which is important for diagnosis and treatment planning.  4. Imaging: If there is suspicion of middle ear pathology, imaging studies such as a computed tomography (CT) scan may be ordered to provide detailed images of the ear structures and identify potential causes of hearing loss, such as cholesteatoma.  5. Microbiological Culture: A sample of the ear discharge may be collected and sent for microbiological culture to identify any causative bacteria or pathogens.  6. Biopsy and Histopathology: In cases where there are abnormalities or if a cholesteatoma is suspected, a biopsy of the tissue may be performed, and histopathological examination can help confirm the diagnosis.  7. Treatment Options:  - Antibiotics: If there is an active infection, antibiotics may be prescribed to treat the infection and manage the otorrhea.  - Surgical Intervention: If a cholesteatoma is confirmed, surgical removal is typically necessary to prevent further complications and improve hearing. This may include tympanoplasty or mastoidectomy, depending on the extent of the cholesteatoma.  - Management of Earwax: If earwax impaction is the cause of the symptoms, the healthcare provider will remove the earwax to alleviate symptoms.  8. Patient Education: The patient will be educated about the underlying condition, treatment options, and the importance of follow-up care.  9. Follow-Up: Regular follow-up appointments will be necessary to monitor the patient's progress and assess the effectiveness of the chosen management strategy.  It's important to note that the exact management plan will depend on the underlying diagnosis. Timely evaluation and treatment are crucial to prevent complications and address the patient's symptoms.  For references and more detailed information, you can refer to reputable medical sources and organizations, such as:  1. American Academy of Otolaryngology-Head and Neck Surgery. (2019). Otitis Media with Effusion Clinical Practice Guideline. [https://www.entnet.org/sites/default/files/uploads/2018-02/OMECPG_FINAL_2-10-19.pdf](https://www.entnet.org/sites/default/files/uploads/2018-02/OMECPG_FINAL_2-10-19.pdf)  2. Chole, R. A. (2017). Cholesteatoma: Diagnosis, Imaging, Techniques, and Complications. Lippincott Williams & Wilkins.  3. National Institute on Deafness and Other Communication Disorders (NIDCD). (2019). Cholesteatoma. [https://www.nidcd.nih.gov/health/cholesteatoma](https://www.nidcd.nih.gov/health/cholesteatoma)  4. Roland, P. S., & Rutka, J. (2019). Practical Management of Cholesteatoma. Thieme.  **Please consult a healthcare professional for a proper evaluation and individualized management plan for the specific condition causing these symptoms**. **]**  **The mean accuracy score of references: 0** | Lawrence R Lustig, MD, Charles J Limb, MD, Marlene L Durand, MD “Chronic otitis media and cholesteatoma in adults”  Text link:  <https://www.uptodate.com/contents/chronic-otitis-media-and-cholesteatoma-in-adults?search=painless,%20foul-smelling%20otorrhea,%20and%20conductive%20hearing%20loss%20on%20the%20left%20side&source=search_result&selectedTitle=3~150&usage_type=default&display_rank=3>  Topic 15312 Version 42.0 |
| *20. Case* | |
| Question: Tell me how would you manage a 1-year-old female patient who comes with otalgia and fever in the right ear for two days and tender mastoid. Give me references at the end of your response.  **[** Answer: A 1-year-old female patient presenting with otalgia (ear pain) and fever in the right ear for two days, along with a tender mastoid, may be showing signs of acute otitis media with mastoiditis, which is an infection of the middle ear that has spread to the mastoid bone. This is a **potentially serious condition, and prompt evaluation and appropriate management are essential**. Here's a general outline of how healthcare providers might manage this case:  1. Medical Assessment: The healthcare provider will begin by assessing the patient's vital signs, including temperature, heart rate, and respiratory rate, and perform a general physical examination.  2. Otoscopy: An otoscopic examination of the right ear will be conducted to assess the ear canal and eardrum. Signs of acute otitis media (e.g., a bulging or red eardrum) may be observed.  3. Audiological Evaluation: In some cases, a hearing test (audiometry) may be performed to assess the extent of hearing loss.  4. Imaging: Imaging studies such as a computed tomography (CT) scan may be ordered to assess the extent of mastoid involvement and identify potential complications.  5. Blood Tests: A complete blood count (CBC) and blood culture may be conducted to assess for infection and determine the appropriate antibiotics for treatment.  6. Medical Management:  - Antibiotics: Empiric antibiotic therapy is typically initiated to treat the suspected infection. Antibiotics effective against common causative pathogens (e.g., Streptococcus pneumoniae, Haemophilus influenzae) are often prescribed. Intravenous antibiotics may be necessary in severe cases.  - Pain Management: Pain relief medications (e.g., acetaminophen or ibuprofen) may be recommended to alleviate otalgia and fever.  7. Inpatient Care: Hospitalization may be required for close monitoring, especially in severe cases of mastoiditis.  8. Surgical Intervention: In certain cases, surgical intervention may be necessary to drain any abscesses within the mastoid bone. This is typically reserved for cases with complications or those unresponsive to medical treatment.  9. Follow-Up: The patient will require follow-up appointments to monitor the progress of treatment and ensure resolution of the infection.  10. Patient Education: Parents or caregivers will be educated on the importance of completing the full course of antibiotics and monitoring the child's symptoms.  Given the age of the patient and the potential seriousness of mastoiditis, prompt evaluation by a pediatrician or otolaryngologist is crucial. Treatment should be tailored to the specific clinical findings and patient's condition.  For references and more detailed information, you can refer to reputable medical sources and organizations, such as:  1. Lieberthal, A. S., Carroll, A. E., Chonmaitree, T., et al. (2013). The Diagnosis and Management of Acute Otitis Media. Pediatrics, 131(3), e964-e999.  2. Lee, J. H., & Kwon, S. Y. (2018). Pediatric Acute Mastoiditis: The Korean Perspective. Clinical and Experimental Otorhinolaryngology, 11(3), 159-164.  3. Marom, T., & Tan, A. (2016). The Otitis Media-6 and 12. Otolaryngology–Head and Neck Surgery, 155(3), NP1-NP3.  **Please consult a healthcare professional for a proper evaluation and individualized management plan for the specific condition affecting this young patient.** **]**  **The mean accuracy score of references: 0.66** | Ellen R Wald, MD “Acute mastoiditis in children: Clinical features and diagnosis”  Text link:  <https://www.uptodate.com/contents/acute-mastoiditis-in-children-clinical-features-and-diagnosis?search=otalgia%20and%20fever%20in%20the%20right%20ear%20for%20two%20days%20and%20tender%20mastoid%20in%20child&source=search_result&selectedTitle=1~150&usage_type=default&display_rank=1>  Topic 6062 Version 29.0 |
| *21. Case* | |
| Question: How would you manage a 49-year-old male patient who comes with dysphonia and difficult breathing for 4 months. Give me references at the end of your response.  **[** Answer: A 49-year-old male patient presenting with dysphonia (voice changes) and difficulty breathing for four months requires a thorough evaluation to determine the underlying cause. These symptoms can be indicative of various conditions affecting the larynx and airway. Here's a general outline of how healthcare providers might manage this patient:  1. Medical History: The healthcare provider will start by taking a detailed medical history, including questions about the onset and progression of dysphonia and breathing difficulties, any associated symptoms, any relevant medical conditions, lifestyle habits (e.g., smoking), and potential occupational exposures.  2. Physical Examination: A physical examination will be conducted, focusing on the head and neck, including the examination of the throat and larynx. The healthcare provider will assess for any visible abnormalities or swelling in the neck.  3. Flexible Laryngoscopy: A flexible laryngoscopy may be performed to visualize the larynx and vocal cords. This procedure is typically done in the office setting and can help identify any structural or functional abnormalities in the larynx.  4. Imaging Studies: Imaging studies, such as computed tomography (CT) or magnetic resonance imaging (MRI) of the head and neck, may be ordered to assess the structures in the throat and airway and identify any possible masses or abnormalities.  5. Vocal Cord Function Testing: If vocal cord paralysis or paresis is suspected, vocal cord function tests may be performed to evaluate the mobility and function of the vocal cords.  6. Voice Assessment: A voice assessment by a speech-language pathologist may be conducted to evaluate the quality and function of the patient's voice.  7. Endoscopy and Biopsy: If there are concerning findings, an endoscopy and biopsy of any suspicious lesions or masses may be performed to determine the nature of the tissue and assess for malignancy.  8. Pulmonary Function Testing: Given the reported breathing difficulties, pulmonary function testing may be ordered to evaluate lung function and capacity.  9. Treatment Options:  - Management will depend on the underlying diagnosis. Treatment may include voice therapy, medication, surgery, or a combination, depending on the specific condition.  - Voice Therapy: In cases of functional dysphonia, voice therapy may be recommended to improve vocal cord function and voice quality.  - Surgery: If a mass or lesion is identified, surgical intervention may be necessary for removal, especially if it is malignant.  - Medical Management: Depending on the diagnosis, medication may be prescribed to address any underlying conditions contributing to the symptoms.  10. Follow-Up: Regular follow-up appointments will be necessary to monitor the patient's progress and assess the effectiveness of the chosen management strategy.  It's important to note that the management plan will be tailored to the specific diagnosis. Dysphonia and breathing difficulties can be caused by various factors, including vocal cord disorders, benign or malignant masses, neurological issues, or inflammatory conditions.  For references and more detailed information, you can refer to reputable medical sources and organizations, such as:  1. Rosen, C. A., Simpson, C. B., et al. (2016). Dysphonia and the Professional Voice. In Bailey's Head & Neck Surgery—Otolaryngology (5th ed.), pp. 1950-1987. Lippincott Williams & Wilkins.  2. National Institute on Deafness and Other Communication Disorders (NIDCD). (2020). Voice. [https://www.nidcd.nih.gov/health/voice](https://www.nidcd.nih.gov/health/voice)  3. American Academy of Otolaryngology-Head and Neck Surgery. (2020). Dysphonia (Hoarseness). [https://www.enthealth.org/conditions/dysphonia-hoarseness/](https://www.enthealth.org/conditions/dysphonia-hoarseness/)  **Please consult a healthcare professional for a proper evaluation and individualized management plan for the specific condition affecting this patient**. **]**  **The mean accuracy score of references: 0.33** | Jean M Bruch, DMD, MD, Dipti V Kamani, MD “Hoarseness in adults”  Text link:  <https://www.uptodate.com/contents/hoarseness-in-adults?search=dysphonia%20and%20difficult%20breathing%20for%204%20months%20in%20adult&source=search_result&selectedTitle=3~150&usage_type=default&display_rank=3>  Topic 6851 Version 48.0 |
| *22. Case* | |
| Question: Tell me how would you manage a 39-year-old female patient who comes with anosmia for 6 days. Give me references at the end of your response.  **[** Answer: A 39-year-old female patient with sudden-onset anosmia (loss of smell) for six days may be experiencing anosmia due to various causes, including viral infections, sinus issues, allergies, or neurological conditions. Here's a general outline of how healthcare providers might manage this patient:  1. Medical History: The healthcare provider will begin by taking a detailed medical history, including questions about the onset and duration of anosmia, any associated symptoms (e.g., nasal congestion, sneezing, fever), recent illness or exposure to viruses, and any relevant medical conditions or medications.  2. Physical Examination: A physical examination may be conducted to assess the nasal passages, throat, and head and neck region, looking for signs of infection or structural abnormalities.  3. Imaging Studies: In some cases, imaging studies such as a computed tomography (CT) scan of the sinuses may be ordered to assess for sinusitis or nasal polyps.  4. Nasal Endoscopy: A nasal endoscopy is often performed to examine the nasal cavity and assess the condition of the nasal mucosa and the olfactory region.  5. Allergy Testing: If allergies are suspected, skin prick tests or blood tests (serum-specific IgE) may be performed to identify potential allergens triggering anosmia.  6. Viral Testing: In cases of sudden-onset anosmia, a reverse transcriptase-polymerase chain reaction (RT-PCR) test for viruses such as SARS-CoV-2 (COVID-19) may be conducted, as anosmia is one of the symptoms associated with this viral infection.  7. Management:  - If a viral infection is identified, management may involve supportive care and addressing the viral infection itself.  - In cases of sinonasal conditions such as sinusitis or nasal polyps, medical treatment with antibiotics, corticosteroids, or surgical intervention may be considered.  - For allergic rhinitis, allergen avoidance, antihistamines, and intranasal corticosteroids may be recommended.  - In certain cases, when anosmia is associated with neurological conditions, the patient may be referred to a neurologist for further evaluation.  8. Patient Education: The patient will be educated about the underlying condition, management options, and the importance of follow-up care.  9. Follow-Up: Regular follow-up appointments will be necessary to monitor the patient's progress and assess the effectiveness of the chosen management strategy.  It's crucial to address anosmia as it can impact the patient's quality of life. The specific management plan will depend on the underlying cause, which can vary widely.  For references and more detailed information, you can refer to reputable medical sources and organizations, such as:  1. Hummel, T., Whitcroft, K. L., Andrews, P., et al. (2017). Position paper on olfactory dysfunction. Rhinology Journal, 54(1), 1-30.  2. American Academy of Otolaryngology-Head and Neck Surgery. (2020). Loss of Smell. [https://www.enthealth.org/conditions/loss-of-smell/](https://www.enthealth.org/conditions/loss-of-smell/)  3. Hopkins, C., & Philpott, C. (2016). Defining the Microbiological and Anatomical Risk Factors for Olfactory Disease: Clinical and Preclinical Evidence. Clinical Otolaryngology, 41(1), 3-9.  **Please consult a healthcare professional for a proper evaluation and individualized management plan for the specific condition causing anosmia in this patient**. **]**  **The mean accuracy score of references: 0.66** | Denis Lafreniere, MD “Taste and olfactory disorders in adults: Evaluation and management”  Text link:  <https://www.uptodate.com/contents/taste-and-olfactory-disorders-in-adults-evaluation-and-management?search=anosmia&source=search_result&selectedTitle=1~128&usage_type=default&display_rank=1>  Topic 6846 Version 46.0 |
| *23. Case* | |
| Question: Tell me how would you manage a 13-year-old male patient who comes with recurrent epistaxis and unilateral nasal obstruction. Give me references at the end of your response.  **[ I'm not a doctor**, but I can offer some general guidance for a case like this. If a 13-year-old patient presents with recurrent epistaxis (nosebleeds) and unilateral nasal obstruction with a septum deviation, here's how it might be managed:  1. Assessment: The first step is a thorough evaluation of the patient's medical history, including the frequency and severity of nosebleeds, and a physical examination, which may include an inspection of the nasal passages.  2. Management of Epistaxis: - Direct pressure: Teach the patient or their guardian how to apply direct pressure to stop nosebleeds. - Nasal saline: Recommend saline nasal sprays to keep the nasal passages moist and reduce the risk of nosebleeds. - Topical agents: In some cases, a healthcare professional may recommend using topical agents to control bleeding.  3. Septum Deviation: - Referral: If the septum deviation is significant and causing symptoms, the patient may be referred to an ear, nose, and throat (ENT) specialist for evaluation. - Surgical correction: In some cases, surgical intervention, like a septoplasty, may be considered to correct the septum deviation.  4. Education: Provide education on proper nasal care and measures to prevent further nosebleeds.  5. Follow-up: Schedule follow-up appointments to monitor the patient's progress and adjust the treatment plan as needed.  References for detailed clinical management and guidelines can be found in medical textbooks, particularly those focusing on otolaryngology, or from medical organizations like the American Academy of Otolaryngology-Head and Neck Surgery (AAO-HNS). Consultation with a healthcare professional is essential for personalized diagnosis and management in such cases. **]**  **The mean accuracy score of references: 0** | Neil Bhattacharyya, MD, FACS “Nasal obstruction: Diagnosis and management”  Text link:  <https://www.uptodate.com/contents/nasal-obstruction-diagnosis-and-management?search=pediatric%20nasal%20obstruction&source=search_result&selectedTitle=7~141&usage_type=default&display_rank=7>  Topic 14609 Version 40.0  Anna H Messner, MD “Management of epistaxis in children”  Text link:  <https://www.uptodate.com/contents/management-of-epistaxis-in-children?search=pediatric%20epistaxis&source=search_result&selectedTitle=1~150&usage_type=default&display_rank=1>  Topic 6484 Version 30.0 |
| *24. Case* | |
| Question: Tell me how would you manage a 2-year-old female patient who comes with a fever, trismus, limited cervical neck extension, and dyspnea. Give me references at the end of your response.  **[I'm not a doctor**, but I can offer some general guidance for a case like this. If a 2-year-old patient presents with fever, trismus, limited cervical neck extension, and dyspnea, it could be indicative of various conditions, including possible serious infections like epiglottitis or meningitis. Immediate medical attention is crucial. Here are some general steps that might be taken:  1. Ensure ABCs (Airway, Breathing, Circulation): Ensure the child's airway is open and assess breathing. If there are signs of respiratory distress, oxygen and possibly intubation may be necessary.  2. Stabilization: Keep the child as calm and comfortable as possible, as agitation can worsen symptoms.  3. Fever management: Administer antipyretic medication like acetaminophen to reduce fever.  4. Consultation: Consult a pediatric specialist or infectious disease specialist for further evaluation and guidance.  5. Appropriate tests: Depending on clinical suspicion, tests like blood cultures, lumbar puncture, and imaging may be necessary.  6. Isolation: If infectious causes are suspected, consider isolation precautions to prevent the spread of the disease.  Please note that this is just a general guideline, and the specific approach would depend on the child's clinical presentation and the doctor's assessment. Always consult with a healthcare professional for a case like this.  For references, consult pediatric textbooks and guidelines like those from the American Academy of Pediatrics (AAP) or the Centers for Disease Control and Prevention (CDC).**]**  **The mean accuracy score of references: 0** | Ellen R Wald, MD “Retropharyngeal infections in children”  Text link:  <https://www.uptodate.com/contents/retropharyngeal-infections-in-children?search=trismus%20children&source=search_result&selectedTitle=8~125&usage_type=default&display_rank=8>  Topic 6078 Version 23.0 |
| *25. Case* | |
| Question: Tell me how would you manage a 28-year-old male patient who comes with preauricular, intermittent, sharp pain, limited jaw motion, and clicking of the temporomandibular joint. Give me references at the end of your response.  **[** Answer: A 28-year-old male patient presenting with preauricular (in front of the ear), intermittent, sharp pain, limited jaw motion, and clicking of the temporomandibular joint (TMJ) may be experiencing temporomandibular joint disorder (TMD). Management typically involves a combination of conservative treatments and, in some cases, referral to specialists. Here's a general outline of how healthcare providers might manage this patient:  1. Medical History and Examination: The healthcare provider will begin by taking a detailed medical history, including questions about the onset, duration, and nature of symptoms, any recent injuries, dental history, and any habits like teeth grinding (bruxism). A physical examination will be conducted to assess jaw movement, muscle tenderness, and TMJ sounds.  2. Diagnostic Imaging: In some cases, diagnostic imaging such as magnetic resonance imaging (MRI) or computed tomography (CT) scans may be ordered to assess the TMJ and surrounding structures for any structural abnormalities or joint displacement.  3. Conservative Management:  - Pain Management: Non-steroidal anti-inflammatory drugs (NSAIDs) or muscle relaxants may be prescribed to alleviate pain and inflammation.  - Dietary Modifications: A soft diet and avoiding hard or chewy foods can help reduce stress on the TMJ.  - Oral Appliances: A custom-fitted oral splint or bite guard may be recommended to reduce teeth grinding and clenching, especially if bruxism is a contributing factor.  - Physical Therapy: Specific exercises and physical therapy techniques may be prescribed to improve jaw mobility and reduce pain.  4. Behavioral and Lifestyle Changes:  - Counseling and education regarding behaviors that may exacerbate TMD, such as teeth grinding, nail biting, and gum chewing.  - Stress management techniques to reduce clenching and grinding related to stress.  5. Referral to Specialists:  - In cases where conservative measures are not effective or if there are structural issues with the TMJ, the patient may be referred to a specialist, such as an oral and maxillofacial surgeon or a TMJ specialist.  - Surgical interventions, such as arthrocentesis, arthroscopy, or joint replacement, may be considered in severe or refractory cases.  6. Patient Education: The patient will be educated about the condition, management strategies, and the importance of adhering to treatment recommendations.  7. Follow-Up: Regular follow-up appointments will be necessary to monitor the patient's progress and adjust treatment as needed.  TMD can have various causes, including muscle tension, joint inflammation, and structural issues within the TMJ. The specific management approach will depend on the underlying diagnosis and the patient's response to treatment.  For references and more detailed information, you can refer to reputable medical sources and organizations, such as:  1. American Academy of Otolaryngology-Head and Neck Surgery. (2021). Temporomandibular Joint (TMJ) Disorders. [https://www.enthealth.org/conditions/temporomandibular-joint-tmj-disorders/](https://www.enthealth.org/conditions/temporomandibular-joint-tmj-disorders/)  2. Kim, M. R., Kim, S. G., et al. (2018). Analysis of Clinical and Magnetic Resonance Imaging Outcomes After Arthrocentesis of the Temporomandibular Joint: A Retrospective Study of 173 Asian Patients. Journal of Oral and Maxillofacial Surgery, 76(3), 481-487.  3. UpToDate. (2021). Temporomandibular Joint Dysfunction (TMD). [https://www.uptodate.com/contents/temporomandibular-joint-dysfunction-tmd](https://www.uptodate.com/contents/temporomandibular-joint-dysfunction-tmd)  **Please consult a healthcare professional for a proper evaluation and individualized management plan for the specific condition affecting this patient's temporomandibular joint**. **]**  **The mean accuracy score of references: 0** | Noshir R Mehta, DMD, MS, David Keith, BDS, FDSRCS, DMD “Temporomandibular disorders in adults”  Text link:  <https://www.uptodate.com/contents/temporomandibular-disorders-in-adults?search=temporomandibular%20joint%20disorder&source=search_result&selectedTitle=1~103&usage_type=default&display_rank=1>  Topic 5629 Version 47.0 |

**Notes:** 1. **[ ]** – *These symbols in the table mean that we copied the text from ChatGPT for each answer without editing/adding our comments.
2. Please consider that references in the table are related to ChatGPT, not to the authors of this paper.*
